# CIRSE Clinical Practice Manual

**DOI:** 10.1007/s00270-021-02904-3

**Published:** 2021-07-06

**Authors:** Andreas H. Mahnken, Esther Boullosa Seoane, Allesandro Cannavale, Michiel W. de Haan, Rok Dezman, Roman Kloeckner, Gerard O’Sullivan, Anthony Ryan, Georgia Tsoumakidou

**Affiliations:** 1 Clinic of Diagnostic and Interventional Radiology, Marburg University Hospital, Baldingerstrasse, 35043 Marburg, Germany; 2 Department of Vascular and Interventional Radiology, University Hospital of Vigo, Vigo, Spain; 3 Department of Radiological Sciences, ‘Policlinico Umberto I’University Hospital, Rome, Italy; 4 Department of Radiology, Maastricht University Medical Center, Maastricht, The Netherlands; 5 Clinical Institute of Radiology, University Medical Centre Ljubljana, Zaloska 7, 1000 Ljubljana, Slovenia; 6 Faculty of Medicine, University of Ljubljana, Vrazov trg 2, 1000 Ljubljana, Slovenia; 7 Department of Diagnostic and Interventional Radiology, Johannes Gutenberg-University Medical Center, 55131 Mainz, Germany; 8 U.C.H. Galway, Interventional Radiology, Galway, Ireland; 9 University Hospital Waterford and Royal College of Surgeons in Ireland, Waterford, Ireland; 10 University Hospital of Lausanne, Lausanne, Switzerland

**Keywords:** Clinical practice, Interventional radiology, Quality standards, Patient care, Practice development

## Abstract

**Background:**

Interventional radiology (IR) has come a long way to a nowadays UEMS-CESMA endorsed clinical specialty. Over the last decades IR became an essential part of modern medicine, delivering minimally invasive patient-focused care.

**Purpose:**

To provide principles for delivering high quality of care in IR.

**Methods:**

Systematic description of clinical skills, principles of practice, organizational standards and infrastructure needed for the provision of professional IR services.

**Results:**

There are IR procedures for almost all body parts and organs, covering a broad range of medical conditions. In many cases IR procedures are the mainstay of therapy, e.g. in the treatment of hepatocellular carcinoma. In parallel the specialty moved from the delivery of a procedure towards taking care for a patient’s condition with the interventional radiologists taking ultimate responsibility for the patient’s outcomes.

**Conclusions:**

The evolution from a technical specialty to a clinical specialty goes along with changing demands on how clinical care in IR is provided. The CIRSE Clinical Practice Manual provides interventional radiologist with a starting point for developing his or her IR practice as a clinician.

## Clinical skills

### Principles of Clinical Care

In 2008, leaders of 42 different societies representing more than 10,000 interventional radiologists came together to create a document that would provide a unified definition of the discipline and its clinical pathways. The result of their efforts was the “Global Statement Defining Interventional Radiology”. This consensus statement declares that interventional radiology (IR) is a medical specialty that focuses on the diagnosis, treatment, and clinical management of patients using minimally invasive procedures guided by medical imaging [1, 2].

This statement provides the foundation for the involvement of IR in the whole process of patient care, and for safely and effectively delivering interventional procedures:

1. All radiologists who provide diagnostic or interventional radiology services to patients should be appropriately qualified and involved in continuing professional development, showing expertise in:
  diagnostic imaging and radiation safety
  image-guided minimally invasive procedures and techniques as applied to multiple diseases and organs
  the evaluation and management of patients suitable for image-guided interventions included in the scope of interventional radiology practice
  continual invention and innovation of new techniques, devices, and procedures.
2. The interventional radiologist should provide patient-centred care, making sure that the patient is at the centre of everything they do. In this care model, patients are in control when it comes to making decisions about their own care and treatment [3].
3. The interventional radiologist should take primary clinical responsibility for the patients they treat, since they are the clinicians best suited to inform, explain, and advise about the procedures they perform [2]. Assuming clinical responsibility means that interventional radiologists should be able to:
  Inform patients of the spectrum of therapeutic options that might benefit them and can be provided by IR, while ensuring that patients have sufficient information to give their fully informed consent for an IR procedure
  Implement the most appropriate management plan after meticulous patient evaluation and determination of the appropriateness of the procedure
  Admit patients to the IR service and provide care before and after therapeutic interventions.
  Provide longitudinal patient care in an outpatient setting not only in the pre-procedure period, but also in the post-procedure period, to assess outcomes, recurrence or development of new problems [4].
4. Effective teamwork and communication with the referring physician and relevant specialists are essential for the delivery of safe and high-quality patient care. Interventional radiologists must seek proper consultation when managing complex cases or when expertise in managing specific conditions is required [5].
5. To deliver effective patient care, interventional radiologists require appropriate clinical time, infrastructure, and support from their employing organisations, including access to outpatient clinics and inpatient beds. Inadequate health care resources, including staffing and equipment, can have a negative impact on patient outcomes [4].
6. Interventional techniques are now at the forefront of the management of many life-threatening emergencies. Access to a robust 24/7 IR cover should be a priority for all acute hospitals.
7. All patients should have timely access to the most appropriate IR procedure, undertaken by an appropriately trained interventional radiologist.

Providing excellent clinical care is as important to the practice of IR as achieving technical success in procedures. Patient care before and after an interventional procedure is equally important as the procedure itself. For this purpose, interventional radiologists require appropriate time, infrastructure and support from their employing organisations.

### Interventional Radiologist: A New Clinical Specialist

Interventional radiologists provide solutions for a broad range of medical conditions. There is hardly any area of medicine where IR does not have some impact on patient management. As a clinician, the interventional radiologist needs to take care of the patient, not of the procedure. It is crucial for an interventional radiologist to assess and follow-up on the patient’s entire medical condition rather than focussing only on a post-procedural assessment [5]. To further expand the role of the IR physician, IRs have to take primary responsibility for the patient. Therefore, the interventional radiologist needs to be competent in basic clinical skills in general medicine. This role also requires a great deal of effort and collaboration with different stakeholders in the healthcare system.

A multidisciplinary network of consulting physicians is needed when interventional radiologists take on primary responsibility for the patient, as every patient is unique and care should not depend on the individual skills of any single specialist. Receiving advice, counsel and a treatment plan from a broad range of medical perspectives on a multidisciplinary team (MDT) will provide the highest quality of care. With IR being part of a multidisciplinary approach, patients will most likely receive the best treatment options for their specific disease or condition. To achieve this, interventional radiologists must not only possess an in-depth knowledge of the least invasive treatments available, but they must also understand other disciplines and innovative procedures to enhance patient outcomes and support the overall treatment plan. In order to be an accepted clinical partner, interventional radiologists need to understand the patient beyond the distinct condition that initially required an IR solution. The importance of seeking proper consultation when managing complex cases has to be emphasised. To this end, appropriate communication between the interventional radiologist, the referring physician, relevant specialists and the patient is key [4, 6, 7].

The use of interventional radiologists as technicians is a concept that should be relegated to the past and replaced with recognition of interventional radiologists as clinicians and partners in delivering modern, high-quality, multidisciplinary team-based patient care.

Interventional radiologists need to assume primary responsibility for management of the patient and his or her condition.

### Skills Required of an Interventional Radiologist

Clinical care is fundamental for achieving the best outcome of any IR procedure. To achieve this goal, appropriate clinical skills beyond the appropriate technical performance of the procedure itself are necessary. Although IR is far more clinically oriented than in the past, resident training at most institutions has not changed, and the development of clinical skills is often not adequately addressed.

The essential clinical skills required for interventional radiologists are not unique to IR and are similar to the other medical specialties that utilise interventional procedures. Therefore, IR should be practiced like any other discipline that provides clinical care. Essential clinical skills that are required from an interventional radiologist include:

1. Acting as a clinician
  Interventional radiologists should act as a clinician. In addition to performing interventional procedures, interventional radiologists should perform ward rounds, inpatient consultations, do outpatient clinics and take part in MDT meetings. These activities take time and effort, but increase credibility and knowledge, which improves patient care.
2. A broad knowledge base
  Interventional radiologists need a knowledge base beyond disease pathology and interventional treatments. They need to be well informed about medical and surgical treatment options, as well as on medical management of common comorbidities, such as diabetes or arterial hypertension. As IR curriculums vary between different European countries, a universal knowledge base is hard to define. CIRSE has issued curricula on IR and interventional oncology that may serve as a minimum requirement for the basic knowledge any interventional radiologist should possess [8, 9].
3. Communication skills
  Radiologists historically have had suboptimal training in in-person communication skills, due to the low exposure to personal interaction, particularly with patients. Good communication skills are indispensable to any clinical specialty, and effective communication improves medical outcomes and patient satisfaction while reducing the risk of medical errors and malpractice lawsuits [10–13].
4. Managing medical therapy
  Interventional radiologists should be able to independently manage the relevant patient therapy. Especially important is the management of anticoagulation therapy [14], peri- and post-procedural antibiotic therapy [15] and risk factors for post-contrast acute kidney injury (PC-AKI) [16, 17].
5. Providing pain management
  Administration of analgesia and sedation in the interventional radiology suite is necessary during painful procedures. Optimal peri- and post-procedural pain management improves patient experience and patient compliance [18–20].
6. Providing outpatient services
  An IR outpatient office permits the participation in clinical patient management in virtually every medical specialty. It is a basis for the provision of continuous patient care. Moreover, an outpatient office is an entry point and a place for examination and education of the patient before and after any interventional procedure [21].
7. Joining MDT meetings
  Interventional radiologists should be permanent members of MDTs and not just be consulted as a “technician”. MDT meetings serve as a patient referral base and build up the reputation and perception of IR as a clinician.
8. Issuing drug prescriptions
  Most radiologists and interventional radiologists do not issue drug prescriptions themselves. However, issuing prescriptions for post-procedure pain management, antiplatelet drugs or antibiotics is necessary for providing continuous patient care.

These skills need to become part of the training of an interventional radiologist, and future curricula for IR should cover these topics.

Interventional radiologists should act as clinicians. The clinical skills required are similar to those of other medical specialties that engage in interventional procedures. Appropriate clinical skills complement the technical aspects of any interventional procedure and improve medical outcome as well as patient satisfaction.

## Principles of IR Practice

### Defining IR Practice

**Fig. 1:**
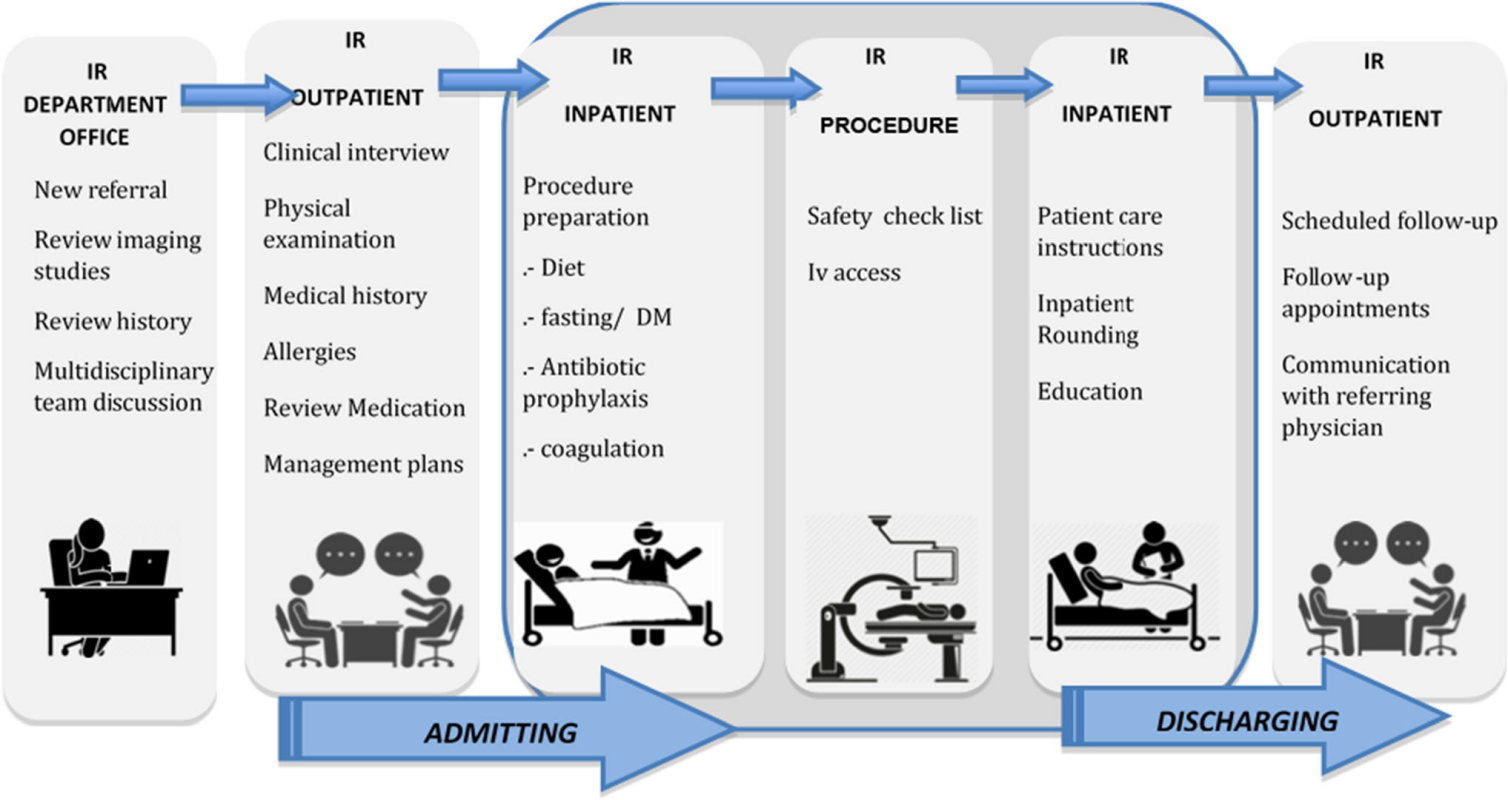
The IR process with the interventional radiologist taking responsibility for a patient through the entire clinical process

IR has evolved from a subspecialty that was, for a long time, focussed on performing specific procedures ordered by various specialists. The development of IR has overcome this model, and a modern IR practice should be a clinical consultative process, whereby a patient is referred (or self-refers) to an interventional radiologist who subsequently directs all aspects of the patient’s care, including reviewing, examining, investigating, devising and enacting a management plan and arranging follow-up. The interventional radiologist provides longitudinal care until the patient’s condition is resolved or kept in abeyance, so that they no longer require IR input and may be referred back to their original physician (Fig. 1). The hallmark of this practice model is that the IR is the clinician with the ultimate responsibility for the patient’s outcomes. This model of care is most clearly demonstrable where physicians external to one’s hospital (e.g. family practitioners) refer directly to the interventional radiologist who has an established IR outpatient clinic (IROC) and day-case/inpatient admitting rights.

Where the older model without admission rights pertains, the opportunity still exists for the interventional radiologist to behave in a more clinical fashion, personally reviewing the patient before scheduling the proposed procedure. If the patient is an outpatient, they can be reviewed in an IR clinic, following which correspondence may be sent to the referring doctor, copying in the family/general practitioner (GP). Over time, this has the effect of modifying other clinicians’ expectations, and is a type of “marketing” to GPs, who can begin to see the interventional radiologist on an even footing with other physicians, paving the way for direct GP referrals. Establishing this mode of practice can be useful in negotiations for additional resources and admission rights with hospital management.

Development and execution of a modern IR process can quintessentially be described in a step-by-step approach as follows:

1. Managing referrals
  When building one’s practice, one should make it as easy as possible for GPs and external hospital consultants to refer, streamlining the process and using electronic means where possible. If possible, a specific “IR consult” code should be introduced by way of “rebranding” (see "Training in IR" section).
2. Pre-procedure planning
  All prior investigations should be reviewed, particularly relevant imaging and previous interventions. The availability of all necessary equipment for the procedure in the department should be confirmed [22]. The use of standard operating procedure (SOP) such as the CIRSE Checklist [23] facilitates pre-procedural planning as well as structured aftercare.
3. Performing the procedure
  A “low hierarchy” culture within the room is recommended to ensure that all members of staff can voice their concerns without fear of rebuke or retribution. The team needs to remain in communication throughout the entire procedure, and particular attention has to be paid to procedural steps that are likely to result in haemodynamic compromise or hyperstimulate the patient.
4. Monitoring and intra-procedural medications
  Minimum monitoring includes: pulse, blood pressure, pulse oximetry and, ideally, a cardiac trace. If sedation is used, capnography is a valuable addition. If an anaesthetist is not involved, patient monitoring needs to be assigned to a person other than the interventional radiologist. The same applies to administration of intra-procedural medications.
5. All prior investigations should be post-procedure and aftercare
  Clear written instructions must be provided regarding monitoring and medications. Potential complications should be clearly flagged and a telephone/beeper number provided to call in the event of any problems. It is crucial that one is approachable, so that the nursing staff feels comfortable sharing concerns [24]. Proper discharge management includes prescriptions, work-absence certificates and a discharge summary to the family practitioner including clear instructions for aftercare and follow-up investigations [5].

In the modern IR process, the IR physician is the clinician with ultimate responsibility for the patient’s outcomes. Even in the absence of inpatient admission rights, behaving more as a clinician than a technician will modify other clinicians’ expectations, and can serve as a type of “marketing” to GPs, who will begin to see the interventional radiologist on an even footing with other physicians. Being accessible with easy referral methods for GPs and self-referring patients is key to a successful IR practice.

### Patient Evaluation and Preparation

The success of any IR procedure depends on the right indication and proper patient evaluation and preparation. This will vary from patient to patient, but the general principles and process steps will be very similar.

#### Consultation

The ultimate goal of the initial consultation is to get a feeling for the patient and a thorough understanding of his or her medical condition. Before or during the consultation, previous imaging studies are reviewed in order to ensure that the appropriate procedure is selected. The interventional radiologist obtains a thorough medical history of the current condition and general medical/surgical status including current medical treatments. The history is then directed towards the patients presenting problem. A directed physical examination is performed to evaluate the patient’s status, level and origin of symptomatology and to adapt the treatment strategy accordingly. Special attention is paid to the risk factors for PC-AKI, allergic predispositions for contrast media, local anaesthetics and antibiotics and current anticoagulation/aggregation therapies. When appropriate, accepted classification systems are used to document and quantify symptomatology (i.e. Visual Analogue Scale (VAS) for pain, International Prostate Symptom Score (IPSS) for lower urinary tract symptoms, the Rutherford and Fontaine symptom classification for extremity ischaemia, etc.).

Lastly, the operator explains the procedure and its benefits in detail to the patient (or his legal representative), informs of the possible adverse events and complications, responds to any questions raised, presents possible therapeutic alternatives and obtains a written informed consent. In all circumstances, a sufficient time between obtaining informed consent and the procedure is required. This interval may vary, depending on the type and risk profile of the procedure. In emergency situations, an exception to the informed consent requirement may be made to prevent serious injury or death or to alleviate suffering (see “Medico-Legal Aspects of IR” section).

#### Pre-procedural Laboratory Testing

Pre-procedural testing can detect any abnormality and allows the interventionalist to either correct it or to adjust/cancel the procedure in order to minimise the risks and avoid complications. Furthermore, it provides a baseline for follow-up monitoring.

Routine laboratory testing before IR procedures includes coagulation profile (prothrombin time (PT), international normalised ratio (INR), activated partial thromboplastin time (a-PTT), platelet count), blood cell count, haemoglobin and renal function. More selective testing is directed according to the patient’s profile and specific procedure. For example, liver function tests (ASAT/ALT, bilirubin, albumin, INR) are needed before liver procedures such as chemoembolisation, liver ablation or biliary drainage. Renal function profiles including creatinine and estimated glomerular filtration rate (eGFR) are recommended for all procedures requiring intravascular administration of iodinated contrast media in order to estimate the risk of PC-AKI [16, 17].

#### Pre-procedural Imaging

Pre-procedural imaging is the decisive factor for indicating an IR procedure. Along with the patient’s history, symptomatology, general status and pathology, pre-procedural imaging defines the type of IR procedure. Imaging should be recent and of sufficient quality. Whenever needed, the operator should order additional imaging before treatment. The interventional radiologist will plan the intervention according to the most recent pre-treatment imaging, including patient positioning (prone vs. supine), type of analgesia, image guidance, trajectory, necessary devices, etc. For some type of procedures, dedicated pre-treatment imaging is mandatory, e.g. planning arteriography with Tc-99 m MAA mapping prior to liver radioembolisation.

#### Pre-procedure Anticoagulation Recommendations

The management of patients receiving anticoagulation and antiplatelet therapy undergoing image-guided interventions is complex and evolving. In clinical practice, there is a lot of variation and no clear consensus exists, mainly due to the lack of evidence-based data [25–27]. For managing anticoagulation in patients undergoing IR procedures, the procedures are stratified according to the inherent “bleeding risk” (Table 1). In addition, the type of medication taken by the patient is considered, resulting in a recommendation (Table 2). Each IR clinic should have an institutional anticoagulation guideline.

**Table 1:** Proposed classification of IR procedures according to the bleeding risk

Low bleeding risk	Moderate bleeding risk	High bleeding risk
Pleural drainage Ascites drainage Superficial drainage Superficial aspiration/biopsy (thyroid, breast, superficial lymph node) Catheter exchange (biliary, nephrostomy, abscess drainage) IVC filter placement Venography Dialysis access interventions	Abdominal biopsy/drainage (except liver, kidney, spleen) Gallbladder drainage Gastrostomy Exchange of biliary tree drain Angiography (access up to 7F) Chemoembolisation/radioembolisation Transjugular liver biopsy Uterine fibroid embolisation Spinal procedures (vertebroplasty, kyphoplasty, lumbar puncture, epidural injection, etc.)	Liver, kidney, spleen biopsy/drainage Biliary interventions Thermal ablation procedures Nephrostomy TIPS

**Table 2:** Example recommendations for the management of anticoagulation and platelet-aggregation blocker therapy before an IR procedure

Bleeding risk category	When to withhold		
	Low	Moderate	High
Aspirin low dose	Do not withhold	Do not withhold	Do not withhold
Aspirin high dose	Do not withhold	5 days	5 days
Clopidogrel	0–5 days	5 days	5 days
Prasugrel	0–5 days	7 days	7 days
Unfractionated heparin iv	1 h	4 h, check aPTT	4 h, check aPTT
Unfractionated heparin sc	4 h	4 h	6 h
Low molecular weight heparin sc	12 h	12 h	24 h
Vitamin K Antagonist, i.e. warfarin	5 days/INR ≤ 2	5 days/INR ≤ 1.5	5 days/INR ≤ 1.5
Dabigatran	24 h	48 h	72 h
Rivaroxaban	24 h	48 h	48 h
Apixaban	24 h	48 h	72 h
Fondaparinux	24 h	36 h	48 h
Acova/Desirudin/Bivalirudin	No	4 h	4 h

In general, the risk from secondary bleeding must be weighed against the risk of complications due to the cessation of anticoagulation or antiplatelet medication. The risks need to be discussed between a relevant physician and the treating radiologist.

For all procedures, regardless of the risk of bleeding, the patient’s platelet count should be at least 50 × 10 ^9^/L. For procedures with low bleeding risk, the INR should be corrected if it is greater than 2. For procedures that have a moderate or high risk of bleeding, the INR should be corrected if it is greater than 1.5. In case of inability or insufficient time to reverse the patient’s anticoagulation status, a reversal agent can be administered; these include vitamin K for warfarin; protamine sulphate for heparin; fresh frozen plasma or platelet transfusion, etc. Some patients receiving long-term anticoagulation may require “bridging” anticoagulation with heparin or low molecular weight heparin.

Critical steps during the pre-procedure consultation include the initial patient assessment, a directed physical examination, the review of the patient’s previous imaging studies and laboratory testing and obtaining written informed consent. Consultations are facilitated by the availability of dedicated institutional guidelines, e.g. for anticoagulation management.

### Peri- and Post-procedural Care

After evaluation, indication and pre-procedural planning, structured peri- and post-procedural care is needed. Typical considerations for peri-and post-interventional care include the following:

#### Sign-in Phase

Interventional radiologists should use a safety checklist (e.g. CIRSE Checklist; see “Quality Management in IR” section) in order to enhance the safety of the procedure by reducing human errors [23]. Prior to any treatment, the interventional radiologist or another member of the IR team involved in the procedure (i.e., nurse, radiology technician) should check that the patient has fasted if needed, that a working peripheral venous access is available and that any anticoagulation or platelet therapy, antibiotic therapy and risk of PC-AKI is managed adequately. The availability of a written informed consent also needs to be checked.

#### Patient Identifiers (“Time-Out”)

Correct identification of the patient and the intervention site and side should be respected in every procedure. If the patient is unable to self-identify, the patient’s relative or companion can do so. If wrist bands are used, they should be attached to the patient at all times and not removed. Failure to identify the patient, to identify the correct anatomic site or the intended intervention can have devastating results.

#### Anaesthesia

Depending on the type and duration of intervention, associated pain, and the patient’s general status and anxiety, an IR procedure can be performed under local anaesthesia alone or in combination with a wide spectrum between conscious sedation and general anaesthesia. While light sedation can be provided by a physician with moderate sedation training, the presence of an anaesthesiology team is mandatory for procedures under deep sedation and general anaesthesia [18].

#### Peri-procedural Antibiotics

The risk of infection in most IR procedures is quite low. Regardless, prophylactic antibiotics need to be administered in specific indications and for selected procedures to diminish the risk of infective complications [28]. Unfortunately, little or no evidence exists in the literature clarifying the need of antibiotic prophylaxis for IR procedures. Nevertheless, antibiotics are important for the peri-procedural management of IR patients, and the interventional radiologist has to be familiar with relevant clinical recommendations [15].

Similar to the surgical literature, IR procedures can be divided into four categories: clean, clean contaminated, contaminated and dirty, each of which is associated with a different risk of infection. According to this classification, most vascular interventions are “clean” procedures and no antibiotic prophylaxis is needed. Nevertheless, in endograft placement procedures the administration of prophylactic antibiotic agents may be recommended, as prosthetic graft infection—though rare—has a high mortality rate. Routine prophylaxis remains controversial in the setting of embolisation for bleeding and solid tumour treatment. Though percutaneous bone procedures are “clean”, the majority of interventional radiologist administer antibiotic prophylaxis, as infectious complications can be difficult to treat.

Procedures involving instrumentation of an obstructed viscus without clinical infection (e.g., biliary or urinary tract obstruction) are typically classified as “contaminated”, and the risk of post-procedural bacteraemia remains high until the organ is adequately drained. Consequently, most radiologists routinely use antibiotic prophylaxis (e.g., third-generation cephalosporin that presents enhanced biliary excretion) for biliary drainage procedures. For genitourinary procedures, it is recommended to use antibiotic prophylaxis in high-risk patients or in those who have signs of infection.

There is no consensus regarding the necessity for prophylactic antibiotics with solid tumour ablation procedures. Many operators recommend the use of antibiotic prophylaxis for liver ablation and sometimes chemoembolisation in patients with biliary-enteric bypass or the presence of biliary stents. In this setting, antibiotics are typically administered at the time of the procedure and continued for at least the following 5–10 days [5, 29].

In general, the antibiotics should be administered just before or at the time of the procedure [30]. Regarding the duration, and according to the surgical experience, one single pre-operative dose is considered to be at least as effective as prolonged treatment [31]. As with anticoagulation management, any IR department should develop and communicate an institutional guideline for the peri-interventional use of antibiotics.

#### Sign-out Phase

In the sign-out phase after the procedure but before the patient leaves the IR suite, a member of the team should verify that any biopsy or other biological specimens have been correctly identified using patient identifiers. The time and date of collection should be documented, ideally including the name of the physician who performed the procedure [32]. Lastly, during this phase, the radiology technician or interventional radiologists should make sure that all relevant images have been correctly uploaded and sent to the Picture Archiving and Communication System (PACS).

#### Post-procedural Analgesia

Depending on the pathology and type of intervention, IR procedures can result in various degrees of post-procedural pain. Minor procedures are, in most cases, correlated with lower levels of pain that can be addressed by non-opioid analgesics. On the contrary, major procedures (i.e. tumour ablation, embolisation, etc.) eventually result in significant prolonged pain. Post-procedural analgesia in these cases usually involves nonsteroidal anti-inflammatory drugs (NSAIDs) and intravenous opioid therapy. The goal of post-procedural pain management is to relieve suffering, achieve early mobilisation and reduce the length of the hospital stay. It is generally recommended to use analgesics in a stepwise pattern, beginning with non-opioid agents, progressing to weak and then strong opioids [5]. Interventional radiologists should be familiar with common analgesics. However, post-procedural pain management can be complicated and, in some cases, beyond the operator’s capabilities. In cases of significant pain not responding to usual regimens, an anaesthesiologist or pain specialist should be involved in post-procedural pain management.

#### Post-procedural Note

At the end of the procedure, the interventional radiologist should provide a brief note, either as an electronic note, a filled-in form or directly in the patient’s chart, where the type of procedure, type of anaesthesia, administered medication, procedure-related details and necessary post-procedural recommendations are listed. Potential complications should be clearly flagged and a telephone/beeper number provided to call in the event any problems. This note should also include recommendations regarding medical treatment, such as post-procedural antibiotics or pain medication. The anticipated length of stay should be documented to facilitate discharge planning. As soon as the patient reaches the ward, this note should be available for the nursing staff.

#### Post-procedural Recovery

Patients should be monitored for varying periods of time, depending on the intervention, after the end of the procedure. The development of any symptoms could indicate the presence of a procedure-related complication. Before patient discharge, written instructions including post-procedural restrictions on activity and diet should be provided to the patient. Finally, for patients that were previously under anticoagulation or antiplatelet therapy clear, instructions need to be provided on when and how to restart the above treatments.

Inpatient rounds should be made regularly for interventions performed on an inpatient basis. A progress note should be kept on the patient’s chart, including patient status, clinical findings, treatment plan and ongoing medication therapies.

Peri-interventional management needs to be structured, including standardised measures for enhancing the safety of the procedure. These measures include, for instance, a pre-procedural “time-out” and institutional guidelines for the use of anticoagulation, antiplatelet therapy and antibiotics. Interventional radiologists have to be able to manage most states of post-procedural pain. A structured post-procedural note is an important tool for documenting and communicating post-procedural recommendations.

### Aftercare and Follow-Up

#### Aftercare

Aftercare and follow-up are integral parts of good clinical practice [33], starting with post-procedure ward rounds. These are most effective when good relationships with ward staff are cultivated, which also helps to ensure that patients receive the prescribed post-procedure care. Nowadays, many IR procedures are performed in day care. Therefore, the follow-up visit is frequently the first moment when the outcome of the procedure can be discussed without the lingering effects of sedation and anxiety of the immediate post-procedural period. It pays to invest in this, because a good doctor–patient relationship is the cornerstone of a successful IR practice and ultimately shows better outcomes and patient compliance.

#### Follow-Up

Follow-up programmes of IR patients show major (inter) national, regional and sometimes even local differences: in some institutes follow-up visits are a structural part of IR care, and in others this is entirely left to the discretion of the referring specialist. Although the underlying logistical, political and/or economic reasons for these differences can easily be understood, well-designed aftercare following structured schedules and protocols (Table 3) lead by an interventional radiologist is of great importance, primarily for IR patients, but also the future of IR as a clinical specialty. Consequently, a schedule for follow-up investigations should also be provided at the time of discharge [5].

**Table 3:** Example for a typical follow-up schedule

	1st day	1 month	3 months	6 month	9 month	12 month	
RFA/TACE Liver tumour		CE MRI/CT	CE MRI/CT	CE MRI/CT	CE MRI/CT	CE MRI/CT	
RFA/Cryo Kidney tumour	US	CE-DUS/CT/MR every 4 months					Every 8 months
Endo Rx PAD		BP, ABI, DUS, Serum lipids	BP, ABI, DUS, Serum lipids			BP, ABI, DUS. Serum lipids	
EVAR		CTA				DUS	

#### Documentation

The importance of proper—objective—documentation of inpatient treatment or an outpatient visit in the patient’s medical record cannot be overestimated [34]. Not only because “if something is not written down, it was never done”, but mainly to substantiate the next steps in the treatment plan and to register intervention outcomes. Furthermore, the referring specialist and/or GP must be informed—in writing—of findings and recommendations after each outpatient visit. Finally, it is of the utmost importance that the patient knows how to contact the IR department at all times for questions about the treatment and to avoid being sent from pillar to post. In addition to the clinical importance of good and clear communication, this confirms the role of the IR physician during the treatment episode. It is indispensable for interventional radiologists to provide discharge summaries and outpatient reports.

A structured aftercare and follow-up programme is important not only for IR patients but also for the evolution of IR into a clinical specialty. Clinical education should be an integral part of general IR training. A well-equipped office is a prerequisite for a successful aftercare and follow-up programme.

### Complication management

Complications are an undesirable but inextricable part of the IR practice. As can be expected from a minimally invasive specialty, the overall complication rate in IR is low. However, a large part of the complications have far-reaching consequences for the patient. Although the importance of complication registration in terms of quality assurance and patient safety is broadly endorsed, there is a reluctance to report medical errors and adverse events. However, complications not only need to be dealt with medically, they also have to be analysed in order to prevent their recurrence [35].

The use of unambiguous, preferably nationally and internationally accepted definitions is an important condition for registering complications. CIRSE states that a complication or adverse event can be defined as “any unfavourable and unintended sign (including an abnormal laboratory finding), symptom, or disease temporally associated with the use of a medical treatment or procedure that may or may not be considered related to the medical treatment or procedure” and suggests that a period of 30 days is applied post-procedurally. National and international uniformity is also desirable for further classification of the complications, and several systems have been proposed, including one by CIRSE [36]. A well-established system for reporting surgical complications is the Clavien–Dindo classification [37]. With such a system, the adverse events can be further analysed and it is possible to compare the data [38]. By mirroring results to, for example, a national average, it is possible for societies or individual radiologists to provide insight into the quality of their own actions and, when possible, to improve it (see “Monitoring of Success” section).

Complications that arise during or immediately after an interventional procedure can often be avoided. Well-known measures to minimise complication rates include careful pre-procedural evaluation, the use of a safety checklist, and effective communication within the interventional team and with the referring specialists. All professionals involved in a patient’s treatment, both during the procedure and in the aftercare process, should be able to identify the sometimes difficult to recognise adverse events in time. This requires specific education, which should be an integrated part of general IR training.

Beyond these basic measures, it is indispensable for a successful interventional radiologist to establish a quality management system. One of the most important tools for managing and preventing complications is the morbidity and mortality (M&M) meeting [39] (see “Quality management in IR” section). Although many endorse the importance of these meetings, they have only been implemented into IR practices to a limited extent [40].

Complication registration is paramount for quality assurance and patient safety. Only internationally accepted definitions should be used when analysing complications. M&M meetings should be a structural part of every IR practice.

## Organisation in IR

### Classification of IR Procedures

There are numerous criteria by which IR procedures may be classified. The classification selected will depend on a defined goal, for example, in medical, training or administrative terms. From a medical perspective, for example, procedures may be classified with respect to the level of difficulty for training purposes, or risk-stratification (e.g. high vs. intermediate vs. low risk of bleeding) [41]. From an administrative perspective, procedures may be classified (coded) for reporting, billing or statistical purposes. Regardless, the classification of procedures and their applications will impact the respective IR process.

Commonly used classifications follow the organ system, e.g. vascular versus non-vascular; pathology—interventional oncology (IO) versus non-IO; purpose—diagnostic vs. therapeutic; guidance method(s) incl. the use of ionising radiation versus guidance without radiation, complexity and urgency. A white paper from the image-guided therapy (IGT) working group of the European Society of Radiology (ESR) proposed a classification based on the underlying condition (IGT in non-tumour conditions vs. IGT in tumours vs. IGT in supportive conditions) [22, 42]. The German Society for Interventional Radiology and Minimally Invasive Therapy (DeGIR) established a modular classification system for training in IR with different levels of complexity. Procedures are grouped as vessel-opening procedures, vessel-closing procedures, non-vascular procedures, oncology procedures and neurovascular procedures [41]. Procedures may be further subdivided into those that the IR can do alone and those for which another clinician is required for a successful procedure, such as anaesthesia.

The classification system most frequently used in a department will depend on factors such as the number of interventional radiologists, their individual skillsets, and the scope of the hospital, e.g., the range of subspecialties and the level of care provided. It is crucial to identify which classes of procedures can be done out of hours, and this information needs to be widely disseminated within the network so that ambulances may divert to a more appropriate hospital when a critically ill patient requires a procedure that cannot be provided locally.

Hospitals may subdivide procedures along lines of cost and place barriers to developing certain procedure sets, such as ablation programmes. In these circumstances, it is helpful to align with other specialty physicians, such as oncologists, and present a joint business case. A hospital’s strategic goals may also be used to support the development of such programmes, for instance, if marketing itself as an “Oncology Centre”, it is self-evident that ablation and chemoembolisation programmes should be supported. It is useful to discuss a subdivision of procedures along lines of cost-effectiveness, for example, IR versus more costly surgical procedures with longer lengths of stay, at a hospital level.

IR procedures may be classified using many different criteria. The use of a particular classification depends on specific goals. In any case, classification of procedures will affect the respective IR process. It is crucial to identify classes of procedures that can be offered out of hours, and this information needs to be disseminated widely within the network.

### Economics in IR

IR has shown enormous growth within the last two decades, with compound annual growth rates of nearly 5%. This development has been driven by significant advances in medical products, which in turn led to an increase in material costs. In parallel, health care providers seek higher revenues, while most health care systems aim at cost-saving. In summary, the economic landscape of IR has changed considerably within this period, making a solid financial calculation indispensable.

#### Current Status

**Fig. 2:**
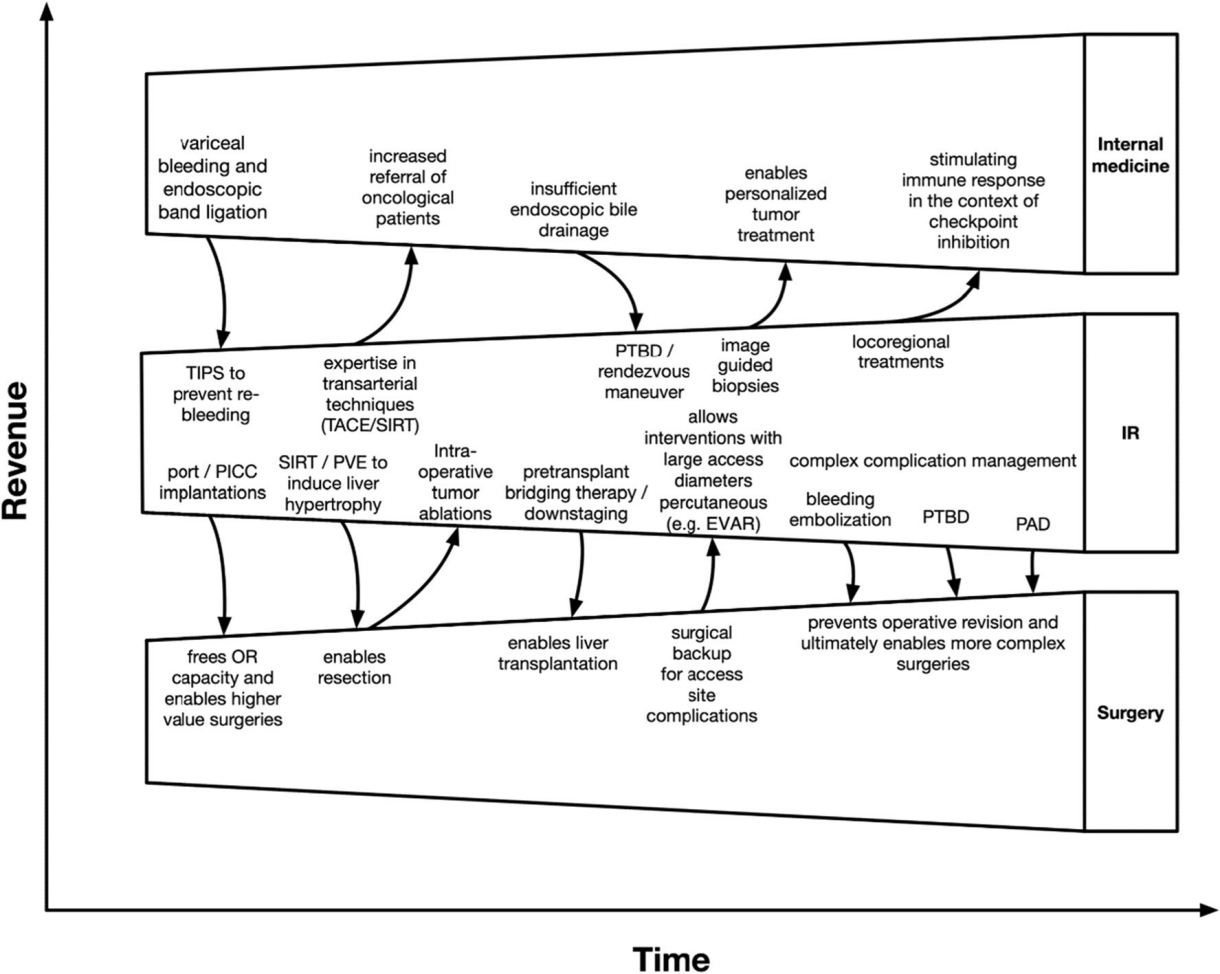
Exemplified win–win situation where IR provides various services for clinical partners, which enables them to increase their portfolio. Examples include TIPS after initial endoscopy to prevent re-bleeding, image-guided tissue sampling, freeing OR capacities for the surgical department by implanting ports or PICC lines, enabling more complex surgical procedures by providing pre- and post-surgical support services, etc. Ultimately, offering such a broad portfolio will increase the number of patients referred to the hospital and specifically attract patients with more complex diseases, leading to a higher case-mix-index so that eventually all departments profit by increasing their revenue

IR plays a key role for several clinical specialties and enables especially the surgical disciplines to perform more complex operations by offering a broad variety of pre- and post-operative techniques. Examples include portal vein embolisation or radioembolisation to induce liver hypertrophy, bridging chemoembolisation prior to liver transplantation, percutaneous biliary drainage, embolisation for bleeding-control before orthopaedic tumour resections, complication management and many more. In turn, IR needs these disciplines as referrers, and sometimes for medical support to solve typical IR-complications. This form of interdependence raises the discussion about financial trade-offs. Possibilities include, for example, fee splitting or internal cost allocation. Interdisciplinary patient management with a strong IR has the potential to increase the total revenue of an interdisciplinary health care provider, by enabling surgeons, for example, to offer more complex operations. Thus, in the end, the financing of an IR department should be seen as a mixed calculation where all clinical partners profit from each other, leading to a win–win-situation (Fig. 2).

#### IR and Cost-Saving

From a payer perspective, IR has the potential to save costs [43]. Firstly, minimally invasive IR procedures are often more cost-effective than their surgical alternatives, for example, liver tumour ablation versus liver resection [44] or port-placement, which was found to be significantly more cost-efficient if performed in an IR-suite than in an operation room [45]. Secondly, minimally invasive procedures might be able to reduce downstream costs by reducing the length of hospital stays or avoiding additional operations (e.g. drainage of abscesses or embolisation of haemorrhages). Thirdly, minimally invasive IR procedures may lead to faster recovery times (e.g. uterine fibroid embolisation vs. surgical hysterectomy) and thus reduce public health costs [43]. However, this may look different from a hospital perspective, as surgical procedures often provide better revenue. These topics represent a typical dilemma between microeconomics (provider perspective) and macroeconomics (payer perspective) with interventional radiologists at its centre.

#### Identifying the Need for IR

Very often, patients who may benefit from an interventional procedure are initially seen when receiving CT or MRI. It is essential to identify such patients at this early stage, to contact the referring physician to offer suitable interventional options and to include this recommendation into the written report [46]. In the case of tumour patients, the interventional radiologist should initiate the discussion in the tumour board. Such immediate actions speed up the entire process considerably and increase the referrer’s satisfaction. If the diagnostic radiologist reading the study is not sure about the indication, he should consult an interventional colleague. Vice versa, interventional radiologists have to communicate with their diagnostic colleagues to ensure that pre- and post-interventional imaging is done using a tailored imaging protocol. Ideally, standard operating procedures should define which type of imaging has to be performed at which point and which communication pathways should be used. All of these steps are cost-efficient and ultimately improve patient care while simultaneously promoting and strengthening IR.

#### Dedicated IR Office

While the organisational details may vary considerably (see “Infrastructure for IR Clinics” section), running a dedicated IR office is essential. It makes it easier to recruit patients and to see them longitudinally, which will strengthen the relationship with the patients. Further, it provides a higher grade of autonomy and facilitates the possibility of seeing outpatients. This will eventually influence the perception of the interventional radiologist not only from a patient’s view, but also from a clinical colleague’s view. They will be seen less as a service provider and more as a clinical peer. Regardless of the organisational details, it is essential to provide an economically sustainable IR practice.

#### Opening an IR Ward

The question of if interventional radiologists should run their own ward is a controversial issue. There are several advantages of running a ward. Firstly, the interventional radiologist will be the primarily responsible physician, providing full service instead of being a service provider. This impacts the patient’s perception of the individual physician and of IR as a whole specialty. Other advantages include a greater flexibility and potentially better reimbursement. However, there are also multiple downsides of running a ward. Only high-volume institutions will succeed in continuously filling an entire ward—if the ward is not fully booked or if the ward is very small, the relative (infrastructural) costs become too high. Furthermore, some referring colleagues might see such an approach as competition, which might negatively influence daily collaboration. An in-between alternative is having access to IR beds in other departments. This enables the interventional radiologist to do their own visits and still provides some flexibility, but frees the IR from the normal duties on the ward. In this type of model, it is crucial to make sure that a relevant part of the revenue is assigned to IR [47]. In summary, there is no good or bad; the optimal concept has to be discussed at each site, ideally in an interdisciplinary fashion so that other disciplines are on board and conflicts can be prevented.

#### Outpatient Versus Inpatient Treatment

From a medical point of view, many interventions that are currently performed as inpatient procedures could also be performed on an outpatient basis. The reasons for not doing so are diverse and include financial and organisational considerations; it may be that the reimbursement is insufficient or that necessary structures, like holding areas for adequate aftercare, are not available [48]. The increasing switch from the femoral to the radial approach may further stimulate this discussion and increase the pressure to perform a higher proportion of interventions in an outpatient fashion [49]. Radiology departments should prepare for this shift towards outpatient treatments.

#### 24-h Interventional Service

Ideally, a 24-h IR-service should be available [46]. However, this can be challenging even in big units due to the limited number of interventionists. To tackle this issue, a sufficiently high number of physicians should be trained to cover at least the most common emergencies like abscess drainages, embolisation of arterial bleeding, etc. To cover also more complex interventions, like emergency transjugular intrahepatic porto-systemic shunt (TIPS) or certain special interventions, a specialised interventional radiologist might be on call. However, this leads to relatively high contingency costs. Still, excellent 24-h complication management is a prerequisite for a variety of complex surgical procedures; these costs have to be appraised as a mixed calculation, and the need depends on the portfolio of the healthcare provider.

#### Business Plan

The leadership of an IR department should write a business plan and revise it on a regular basis [47]. Such a business plan should cover the most relevant aspects of running the IR service, namely the market strategy, an analysis of the competitive landscape, a plan for business development, an operations plan and financial planning. The most important aspect for the IR leadership is the department’s strategy, which needs to be clearly defined and adjusted over time in correspondence with the developments of the department and its hosting organisation. Exemplary questions for business strategy are the overall setting of the IR department, its ownership, its business partners, and its competitors as well as its financing and revenues. A practical and straightforward exercise to develop and refine the IRs business strategy is a so-called SWOT analysis (a 2 × 2 matrix covering the **S**trengths, **W**eaknesses, **O**pportunities and **T**hreats of a business).

**Fig. 3:**
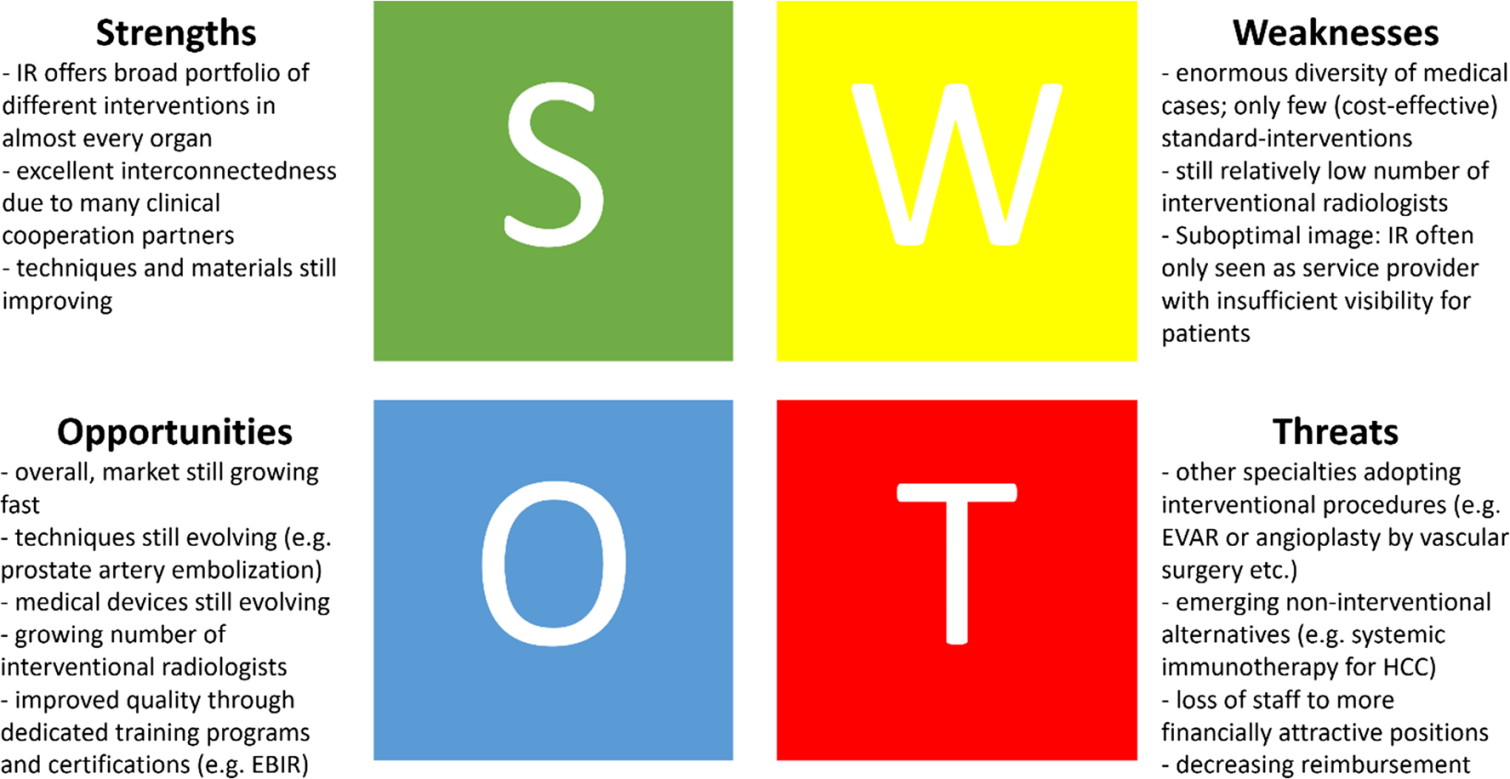
A SWOT analysis covering **S**trengths, **W**eaknesses, **O**pportunities and **T**hreats of a typical IR business on one glance, an essential tool in most business plans

In a typical IR department at an academic tertiary care centre, strengths include the availability of high-end health care, broad interdisciplinary interaction with other medical specialties and the option to perform scientific research, etc. Weaknesses are the enormous diversity of medical cases and a lack of focus on (cost-effective) standard interventions, as well as possible challenges in strategy alignment due to conflicting stakeholder interests, etc. Opportunities are techniques in IR replacing open surgery, the ongoing development and refinement of medical devices, etc. Threats include potential competitors, unwanted attrition of well-trained staff, and development of more effective treatments by other disciplines, etc. (Fig. 3).

#### Controlling

Last but not least, controlling is vital for the business success of an IR department. Since IR departments are most often localised within larger hospitals, controlling can be organised in different ways. It is vital for the IR leadership to know the department’s costs. This includes personnel costs but to the same extent costs for consumables, something many IRs are not aware of. So-called cost-education may raise awareness of this issue and ultimately lead to more cost-effective care [50]. Especially in larger units, the problem of interfaces plays a key role. Often, it is not transparent which department pays which sum of money to which department for a particular service. The resulting financial streams within institutions can reach a remarkable level of complexity. Thus, the central aim should be to develop transparent cost allocation and to break down revenue distributions in terms of a full cost accounting. Finally, a correct invoicing practice with the health care payer is pivotal to redeem appropriate revenues.

The economic landscape of IR necessitates a solid financial calculation. Interventional radiologists need to know the pros and cons of arguments for different business models such as in- and outpatient clinics, IR wards, etc. Knowledge about economic tools, such as performing a SWOT analysis or developing a business plan, is indispensable in modern IR.

### Quality Management in IR

Quality management (QM) is often seen as additional bureaucracy, a burden hampering productivity. Furthermore, it costs time and money, something that is a scarce good in today’s medical world. Nevertheless, QM is not only required to receive various certifications—it is an absolute necessity to further improve quality in an increasingly complex medical environment. Every interventional radiologist can remember multiple so-called near misses situations, which had the potential to result in harm for the patient if the circumstances had been slightly different. Such sentinel events have to be taken seriously and a functioning QM programme is the most effective technique to prevent further serious events from happening. QM programmes vary between institutions, but there are some essential parts presented in this section [51].

#### Standard Operation Procedures (SOP)

SOPs are among the most important methods of quality management in medicine. The International Council for Harmonisation (ICH) defines SOPs as "detailed, written instructions to achieve uniformity of the performance of a specific function". In IR, an SOP is usually a set of step-by-step instructions extensively describing a certain intervention (e.g. chemoembolisation, etc.). SOPs should not only cover the intervention from the view of the interventional radiologist but also include preparation and post-processing done by support staff. Such a “recipe” ensures that all steps are carried out in the correct order and that none are missed. Further, it has the potential to improve coordination and communication within the IR team because everybody knows the workflow and each other’s duties.

SOPs should not be static; they have to be adapted constantly, either to improve them because insufficiencies emerged during daily routines, or to adapt them to a changing situation. Irrespective of that, SOPs have to be revised regularly, usually annually or biannually. Developing a set of SOPs requires a considerable investment of time; however, this effort will usually pay off within the first year [52].

CIRSE offers a broad range of “standards of practice” documents on their website covering the most common interventions. These documents can be used as a blueprint to develop institutional SOPs adapted to the local situation.

#### Checklists

**Fig. 4:**
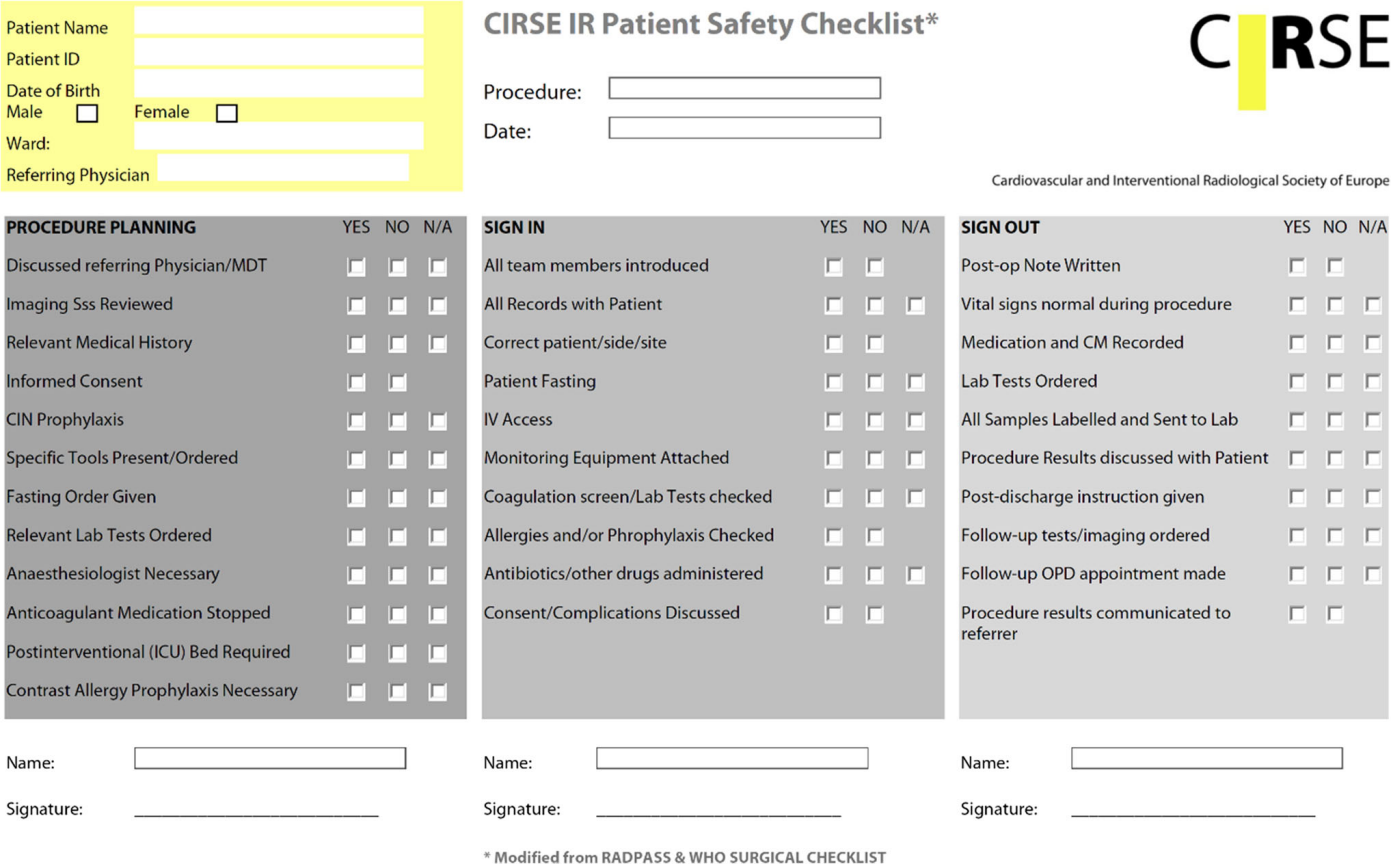
CIRSE patient safety checklist covering all phases of an intervention

Checklists are integral to SOPs. An SOP usually contains several checklists, which can be compiled according to the target audience (e.g. nurses, technicians or interventional radiologists) or the stage of an intervention (preparation, vascular access, etc.). For developing such individual checklists, the target audience should be integrated in the development and implementation process. This is beneficial for quality and completeness and also increases acceptance within the team. Illustrations, such as flowcharts or a photograph of a typical operating table, also increase comprehensibility. An example of a simple checklist is the CIRSE patient safety checklist (Fig. 4) [23]. This dedicated IR checklist was developed by a CIRSE task force ten years ago, and has proven to be effective in reducing the number of critical incidents [53]. However, there still is a need for better dissemination in the IR community.

#### Critical Incident Reporting System (CIRS)

The goal of zero-failure is neither realistic nor reasonable. Instead, every incident has to be considered as a chance to learn and to install measures preventing similar situations from happening in the future. A CIRS is an invaluable tool for learning from critical situations. The first step is to identify and report such critical incidents. A prerequisite for this is to maintain an open quality culture in the department where nobody has to be afraid of sanctions. The next step is to analyse the incident and to develop measures to prevent similar events from happening in the future. This process should ideally include all relevant team members. After approval, these measures have to be openly communicated to the target audience; ideally, the pertinent measures should be implemented into a written document like an SOP. The last step is to monitor whether these measures are effective for preventing future incidents of the same type. CIRS is an appropriate technique for fostering the implementation of a culture where quality matters for everybody to the same extent including interventional radiologists and staff like nurses, technicians, receptionists, etc. [51].

#### Morbidity and Mortality Conferences

As already mentioned in “Complication Management” section, M&M conferences provide clinicians with an opportunity to discuss medical errors and adverse events and are an indispensable tool for quality improvement. Although they were invented more than a century ago and have repeatedly shown their ability to ultimately improve quality of care throughout all specialties, they take place infrequently at most institutions [54]. In a CIRSE-supported survey, less than half of the respondents held regular M&M conferences, although 94% deemed them useful [40]. This willingness to take responsibility is reflected by the great success of M&M sessions, which have been implemented into the schedule of most congresses. With the International Conference on Complications in Interventional Radiology (ICCIR), CIRSE has even introduced a dedicated meeting to the conference schedule. Such sessions at conferences show high attendance rates, but less than half of us have implemented M&M conferences into daily practice is lack of time [40]. Nevertheless, such formats can be of high educative value for all participants, irrespective of their level of experience and their profession; although physicians are the typical target audience, other team members like technicians, nurses, administrative staff, etc., might also profit from such an experience. Typical intervals between M&M conferences range from one to three months, and they should take place on a fixed schedule rather than on-demand. The dates should be determined and communicated to the entire staff several months in advance to facilitate participation. To ensure continuity, one or two staff members should ideally be appointed responsible for organising such events over a longer period. Multidisciplinary staffing is crucial for allowing in-depth discussions from different points of view to maximise the learning effect; at the very least, all professionals who were directly involved in a particular case should attend. M&M conferences should be formalised and stringently organised by a moderator. A typical format starts with a brief case presentation, which can be followed by clarifying questions. The next step is to identify all possible errors, to discuss these in an open manner and eventually devise strategies to prevent such errors from happening again.

#### Communication

Shortcomings in communication are often the root cause of medical errors. This includes insufficient as well as suboptimal communication with all partners at all steps of the procedure. To establish the correct indication for a particular treatment, close communication with the referring physician is mandatory. Most indications will be straightforward, but some will require intense discussion and likely include additional clinical partners. Today, MDT meetings provide additional quality control and are in fact a form of QM. The joint discussion with all relevant experts ensures that treatments are reasonable and prevents wrong indications. Many accreditation authorities explicitly demand repeated pre- and post-procedural presentation in a tumour board for certification as an oncology centre. This guarantees an ongoing quality circle and ultimately improves surveillance and subsequent therapies.

One important interface is the communication between the interventionist and the physician performing further treatment. A dedicated report should accompany every patient when they leave the IR department (see “Peri- and Post-procedural Care” section). This report should contain information at least on what has been done, if complications occurred, and what medication has been administered. Even more important is a section explaining further steps; which type of medication should be given in the future, is additional imaging necessary, do drainages have to be flushed, is there even a need for a follow-up intervention, etc. Such a report can be short and preliminary but should follow a stringent und uniform layout to make it more comprehensible [55].

Alternatively, today’s IT-infrastructure allows for the rapid creation of a (final) report, especially when using dedicated structured reporting (SR) templates instead of free text reports. Compared to free text reporting, SRs are more complete regarding medical content, beneficial for coding efficiency, and significantly increase satisfaction among both interventional radiologists and referring physicians [56].

#### Minimum Volumes

Many medical specialties have already shown that performing a sufficient number of a specific procedure is a prerequisite for quality. Consequently, many specialties have introduced minimum volumes for particular operations, reaching from knee replacements to liver transplantations. According to CIRSE, the minimum number of oncological procedures yields 150 annually, with ideally at least 30 in each subcategory, e.g. ablation, radioembolisation, chemoembolisation, pain management, musculoskeletal interventions, etc. [57]. To ensure that the skill of each interventional radiologist performing such procedures remains at a high standard, a local training plan is mandatory. This could include self-teaching, visiting CME-certified courses or meetings, etc. The practical training may comprise simulator training to a certain amount. The number of staff members is also important, and appropriate staffing is a prerequisite to prevent overwork and maintain high-quality performance.

#### Certifications

Certifications are an effective way to ensure a consistently high quality of care. On an investigator level, the certification of the European Board of Interventional Radiology (EBIR), which is organised by CIRSE, is the most important certification for IR and has recently been endorsed by the UEMS-CESMA (European Union of Medical Specialists—Council for European Specialists Medical Assessment). As of 2020, the group of EBIR titleholders numbers 670 interventional radiologists and is still growing. The examination is a two-step procedure based on the second edition of the European Curriculum and Syllabus for Interventional Radiology [6]. The contents outlined in this document are further specified in the respective CIRSE guidelines (https://www.cirse.org/education/standards-of-practice/). Several national societies offer their own certification programmes, the German Society for Interventional Radiology (DeGIR), for example. This particular programme offers six different modules (A-F), each of them in a basic and an advanced version.

On an institutional level, the whole IR department or even the whole hospital can undergo certification. The International Accreditation System for Interventional Oncology Services (IASIOS) is not only endorsed by CIRSE and the European Cancer Organisation, but also by more than 20 national IR-societies. Several of these national societies also offer own accreditation programmes. In Germany, for example, the DeGIR offers a general accreditation programme covering the whole spectrum of IR. Regarding vascular IR, the DeGIR collaborates closely with the national societies of angiology (DGA) and vascular surgery (DGG), offering a joint certification and fostering interdisciplinary spirit.

QM in IR is more than additional bureaucracy. Establishing a QM system in IR will cost time and money but will soon pay off. Valuable QM tools for IR include SOPs, checklists and M&M meetings. Certifications are an effective way to ensure a consistent quality of care. With deficits in communication being a common root cause for medical errors, particular emphasis has to be put on developing effective communication.

### Marketing in IR

The traditional model of IR has been referrals from other clinicians to interventional radiologists; and in the past, it was primarily a technically based, procedure-related in-hospital service [58, 59]. Experience over the last 20 to 30 years has consistently shown that, over time, other specialties learn the techniques of some of the procedures in which interventional radiologists excel. Although other specialists do not regularly achieve the same level of technical proficiency, they nonetheless control access to the patient, and as a result threaten to marginalise IR [60–62]. To become more independent, it is crucial not only to get direct access to the patient, but to communicate IR’s portfolio to referring physicians, including GPs, and to provide a low-threshold access to IR. Ease of access is key! Marketing is a tool for achieving this goal based on studying short-term and long-term needs of those who can pay for IR services. Marketers (interventional radiologists) can direct their service to other businesses (referring physicians; B2B marketing) or directly to consumers (patients, B2C marketing).

#### Audience

The first step in marketing is the definition of the most relevant target audience. Modern models of IR practice delivery highlight the importance of direct access from patients and GPs to interventional radiologists [63]. Targeting the audience is possibly the single biggest factor in the successful development of a successful IR practice.

To enter the market, interventional radiologists need to behave exactly like other clinicians—except that new marketers need to outperform the other, previously present market actors. For this purpose, interventional radiologists must present a ready-made target for other clinicians to refer patients directly to IR; IR must also be accessible to primary care referrals and from patients themselves.

“Landing” a referral (electronically or by letter/fax) into a large radiology department with several senior radiologists and mostly diagnostic colleagues holds a significant risk for a referral to go astray. Experience has shown that there are few events more irritating for a referring physician than the intended doctor NOT receiving the referring letter. It raises questions about competence, interest, and ultimately whether they would consider such a referral again. The patient is annoyed, and the referring doctor has to WORK to get the attention required. They may do it once or twice, but if it is a recurring pattern it will be the downfall of any interventional radiologist. For ease of access, a dedicated fax line and e-mail referral pathway are recommended. Moreover, interventional radiologists need to get out of their radiology reading rooms and make their presence felt at ward level in the hospital, in GP’s surgeries and in the wider community by means of the internet and social media.

#### In-Hospital IR

Interventional radiologists need to participate in grand rounds, participate in MDT meetings as well as surgical and medical M&M conferences, and to make themselves central to discussions and decisions about treatment care. It is not enough to wait for referrals from other clinicians who may or may not think of IR. Interventional radiologists need to be physically present and involved—or as the phrase goes, “eat or be eaten”. Unless interventional radiologists are actually making decisions about patient care or participating in those decisions, it is very difficult to expect patients to be consistently referred to IR when there are other physicians or other treatment options available. An obvious example is tumour boards with oncology patients; if an interventional radiologist has not attended a MDT meeting, the referral pattern tends to be for biopsies, drainages, etc., and patients are referred for surgery, chemotherapy or radiotherapy. If the IR is physically present at the oncology MDT meeting, the referral pattern changes [64, 65]. Patients are seen by interventional radiologists; a formal consultation takes place and, finally, these same patients are sent to IR not just for adjunctive measures, such as biopsy and drainage, but for the primary therapeutic procedures like tumour ablation, chemoembolisation, etc. The interventional oncology model has been very successful in delivery of this—which has been achieved by attendance at oncology MDT meetings.

It is very important that interventional radiologists are seen on the ward; that the entire ward system, through physicians, nurses, administration, porters, etc., gets used to seeing the interventional radiologist, preferably wearing a white coat with interventional radiology visibly stamped across it—visibility is key. Obviously, this ties in with pre-operative assessment and post-operative ward rounds assessing patient well-being and outcomes after IR procedures. The greater the visibility, the better the target audience can be reached.

#### Marketing to GPs

General practice groups are usually very keen to obtain continuing medical education (CME) and continuing professional development (CPD). These events are well organised, frequently well attended and pose an ideal forum for interventional radiologists. Experience has shown that delivering these learning sessions can result in many patient referrals from GPs. This is primarily about increasing awareness of what interventional radiologists have to offer [66–68].

The following case study may serve as a blueprint for these events: a GP-CME talk at a local restaurant might be titled “Modern Methods of Varicose Vein Treatment” [69], and, at least six weeks in advance, the local representative for compression stockings has been asked by the IR to sponsor it. With every radiology report that the IR department sends out, administrative staff in radiology is asked to insert a brightly coloured flyer mentioning this CME activity. A couple hundred “flyers” are given to the representative, the department and the sales “rep” publicise the event through social media, and junior radiologists in the department tell their friends on the clinical teams. The organising IR goes in person to the restaurant in advance to ensure they serve the food on time, and that there is a good space with a computer compatible projector to present. It is crucial to ensure that the talk is pitched at the right level—not for hospital consultants, not for medical students, not for patients—but for GPs. At the start and end of the presentation, the IR’s email or secretary’s email should be included; this information should also be printed on the flyer, and on the placemats at the restaurant, and business cards should be scattered all over the room.

The talk needs to be aimed at solving GPs problems, which means providing a solution for the problems patients present to their GP. In their practice, a GP generally has six minutes to assess the patients, arrive at a working diagnosis, and either treat or refer onwards. If interventional radiologists provide the solution—even if the GP does not have the diagnosis—the GP will send the patient to an IR. Now, the interventional radiologists are in charge, taking primary responsibility for the patient. That means seeing the patient in the IR outpatient clinic, performing US or MR imaging and whatever tests are needed. It is not critical to know the diagnosis every time—but rather to be the “go-to” doctor for the GP’s problems. The encouraging thing about this entire episode is the number of referrals, not just for varicose veins, but for fibroids, pelvic pain investigation, arterial disease, flank pain, etc. The volume of work that can be generated this way is considerable. So when negotiating the contract it is essential that there is capacity within the job specification to allow for increases in direct referral.

#### Marketing to Patients

Within the hospital, there should be an understanding, professional level of interaction with different consultants and GP colleagues. That means that the other physicians filter out patients with less focussed problems who would be less likely to benefit from IR than from another specialty. This is different with patients who directly refer themselves to IR. The following case study on pelvic pain is a classic example:

When a patient presents herself to an interventional radiologist with pelvic pain, it should be insisted that she see a gynaecologist if she has not already done so. The IR should personally refer the patient to the gynaecologist, with a detailed letter about their symptoms, clinical findings, and imaging results, as well as a mention of potential diagnoses. Even when the diagnosis is likely to be pelvic vein congestion syndrome, if there are urinary incontinence symptoms the possibility that a vaginal hysterectomy and colpo-suspension might be a consideration should be raised, as should the suggestion of a cervical smear, alongside the request for the expert opinion of the other doctor. If the gynaecologist wants to take over care of the patient, then so be it; the IR has done their job—but that gynaecologist knows for certain that they are dealing with a clinician who happens to be very proficient at uterine fibroid embolisation (UFE), pelvic vein embolisation (PVE), ovarian cyst drainage, etc.—rather than just a technician who does a procedure. As a consequence, the gynaecologist will refer back to that IR in the future.

A great deal of attention must be given to patients who self-refer, as these patients have not been pre-filtered by other physicians. The interventional radiologist needs to behave more like a GP who happens to be an expert in a particular sphere, as opposed to an IR specialist who is an expert in one thing only. Self-referring patients are more often female, younger, well-educated, and they arrive with many pages of questions. It is helpful to set a time limit for the consultation before beginning. It is critical to establish a trusting relationship, and more importantly, to establish realistic goals and expectations for any treatment. As this can be seen as a form of marketing, it might be prudent to dampen expectations at the first consultation and “under-promise and over-deliver”, as the patient is likely to tell other patients about the experience. One can be fairly certain that in customer-to-customer (patient-to-patient, C2C) marketing, a detailed account may be uploaded to social media before the clinic is over; and this pattern tends to recur if/when the actual procedure occurs.

The single most important step in marketing IR is to target the proper audience. Strategies differ between in-hospital, GP and direct patient marketing. Low threshold access is as important as the clinical presence and visibility of interventional radiologists.

### Training in IR

Radiology training time typically varies from 4 to 6 years. In the UK, IR training takes an indicative period of 6 years. For the first three years, trainees follow the clinical radiology curriculum, working towards general radiology capabilities. In the following three years, they will build on these general radiology capabilities while developing advanced IR skills to meet the IR specific capabilities set out in the curriculum [70].

#### Curriculum and Training Schedules

The IR curriculum outlines a framework for the process of training and the competencies needed for its successful completion. It is an educational guide to be implemented, interpreted and evaluated by local faculties, radiology schools and local training programme committees. It aims to ensure that interventional radiologists are competent at providing high-quality service for their patients. CIRSE has specifically developed the European Curriculum and Syllabus for “general” interventional radiology, as well as for interventional oncology and for students. There are also national curricula that are balanced with national specifics [41].

Within the IR curriculum, the following competencies will need to be achieved in each disease-specific area. It is desirable to have a stratification of escalating competencies and a formal process of assessing these during training. Capabilities and competencies may be classified and measured (Table 4) as follows:

- Knowledge
- Clinical skills
- Technical skills

**Table 4:** Approach for measuring capabilities and competencies in IR

"Knowledge" competencies will be assessed sequentially for levels as: (1) Knows of; (2) Knows basic concepts; (3) Knows generally; (4) Knows specifically and broadly
"Clinical and Technical skills" may be assessed sequentially for levels as follows; (1) Has observed; (2) Can do with assistance; (3) Can do but may need assistance; (4) Competent to do without assistance, including dealing with complications To achieve level 4, the trainee must be able to work at a level expected from a specialist in the field

Keeping a personal log-book of all performed procedures, including the type and number of procedures and if they were conducted as a second operator, first operator under supervision or as an independent first operator, is strongly advised.

#### Simulators

Similar to surgery, IR is primarily a skills-based specialty. Current training in this field is based on the apprenticeship model, and technical skills have historically been acquired by deliberate practice on patients with guidance from a mentor. It has been suggested that an estimated 10,000 h of procedural practice are required to obtain expertise in such a field. Simulation-based teaching is a widely growing and popular training option among trainees, as has the ability to offer safe practice models for IR training in artificial yet realistic environments. As simulators mainly improve technical skills, their use should be focussed mainly on specific objectives of an IR curriculum [71].

The basis for any successful interventional radiologist is their core competence in IR. This has to be achieved by clinical training, ideally according to a structured curriculum which sets out minimum requirements. While the CIRSE curricula can be used as blueprints for studying, harmonised sources of training content, simulation and structured supervision are key for an effective training in IR.

### Monitoring of Success

Providing a high standard of care is a goal for every medical specialty. To achieve this goal, the interventional radiologist has to monitor the success and assess the quality of IR procedures. This is done by defining performance indicators (PI) for success and complication rates that are generally considered acceptable, and by designing a system for monitoring and evaluating the provided care.

#### Quality Assessment in IR

The goal of monitoring is to identify problems, apply the corrective measures and improve clinical workflow and clinical guidelines. This task is typically part of a quality management programme (see “Quality management in IR” section). It requires continuous data collection, including assessments of clinical success and procedure-related complications. The findings are typically analysed by the use of external and internal benchmarks. Guidelines for establishing a quality assessment programme have been published by the Society of Interventional Radiology (SIR) [72]. The document defines the following steps for designing a quality assessment programme:

1. Form a quality committee
2. Define the provided care and identify important aspects of care
3. Identify indicators related to the important aspects of care and establish the thresholds
4. Collect and organise data
5. Evaluate care when thresholds are reached and take action to resolve identified problems
6. Evaluate the improvement of care and communicate relevant findings

The programme should continuously assess IR procedures performed at a given institution. Data collection should be based on SOPs or “quality improvement guidelines” published by CIRSE, SIR or other related societies, and threshold levels should be compared to published references. When indication and success rates fall below a minimum threshold, or when complication rates exceed a maximum threshold, a review should be performed to determine the causes and implement any necessary changes. A well-working software solution has been established by the DeGIR, where treatments are documented on an institutional basis, but can be analysed at different levels ranging from an institutional analysis for a particular type of procedure to a national level for all types of procedures [73].

#### Performance Indicators

PIs are measurable values that are used to assess progress towards a desired outcome. Clinical indicators are ideally determined by the published evidence. If the evidence in the literature is weak, indicators are often based on professionals’ expert opinions and a consensus process. The most important outcome measures in IR are the success of the procedure and its complication rate. Due to the wide range of different IR procedures, it is virtually impossible to define a single performance indicator and its threshold value valid for all procedures. Each procedure, therefore, has its own specific PIs for the evaluation of success and complication rate. PIs and their threshold values for the most common IR procedures are given below.

#### Percutaneous Needle Biopsy

Indications on percutaneous needle biopsy were published in guidelines from CIRSE in 2017 [74]. The document cites recommended indicators and threshold levels determined by guidelines that the SIR published in 2010 [75]. The success rate threshold level for overall diagnostic biopsy is 75%. This threshold varies depending on the mix of organ systems, lesion locations and histology of the lesion. Consequently, the threshold level should be adjusted by the institution accordingly. The threshold for complications varies widely depending on the biopsied organ. The overall major complication rate should not exceed 2% [75].

#### Percutaneous Nephrostomy

Indications and performance indicators for percutaneous nephrostomy insertion have been determined in a document by SIR [76]. The threshold for technical success in the presence of a dilated collecting system is 96% in a native kidney and 98% in a transplanted kidney. In the event of a non-dilated collecting system or the presence of complex staghorn calculi, the threshold level is at 80%. The most common complications and their thresholds are septic shock (4%), bigger haemorrhage requiring transfusion (4%), vascular injury requiring intervention (1%), pleural complications (1%) and bowel transgression (1%). The relative number of complications that result in transfer to an intensive care unit, emergency surgery, or delay discharge should not exceed 5% [76].

#### Percutaneous Abscess and Fluid Collection Drainage

Indications and performance indicators for percutaneous nephrostomy insertion have previously been summarised by SIR [77]. The technical success threshold for fine needle aspiration of fluid collections is 95%. The threshold for successful abscess drainage (defined as curative or partial improvement) is 85%. The threshold for all major complications resulting from percutaneous abscess drainage is 10%. The threshold for specific major complications is as follows: septic shock (4%), haemorrhage (2%), bowel transgression (2%), pleural transgression (2%) [77].

#### Vascular Access and Closure Devices

A document on vascular closure devices (VCD) recognises the existence of several different VCDs and recommends a proper deployment success rate threshold of 90%, and a successful haemostasis success rate threshold of 90%. Major access site-related complications should not exceed 3% with either manual compression or the use of VCDs [78].

#### Diagnostic Angiography

Guidelines for diagnostic angiography recognise that non-invasive imaging techniques have replaced diagnostic arteriography for many indications and list indications for invasive angiography. When fewer than 95% of procedures are performed for these indications, the department has to review the patient selection process. The complications are divided among access site-related, contrast-related and catheter-induced. Access site-related complications and their thresholds are major hematoma (3%), occlusion (1%) and pseudoaneurysm (0.2%). Contrast-related complications (allergic reactions to contrast, PC-AKI) should not exceed 5%. Catheter-related complications, such as distant embolisation, dissection and subintimal contrast injection, should not exceed 1% [79].

#### Vena Cava Filter Placement

Indications and performance indicators for percutaneous nephrostomy insertion have been determined in a document by CIRSE [80]. The technical threshold for VCF placement should exceed 97%. The thresholds for complications are as follows: IVC occlusion (10%), recurrent PE (5%), filter embolisation (2%), major access site thrombosis (1%) and death (less than 1%). Traceable events can be part of the quality improvement metrics and include IVC penetration, filter movement or fracture, recurrent pulmonary embolism, access site thrombus, IVC occlusion, and insertion problems.

Monitoring of success is vital for providing a high standard of care. A quality assessment programme should be integrated into the departmental workflow to permit continuous data collection. The data need to be continuously analysed using PIs with threshold values. Actions should be taken if the threshold is reached.

### Radiation Protection

The number and complexity of fluoroscopy- and CT-guided interventions have increased over the past 20 years, resulting in higher radiation exposure for patients and staff. During a complex interventional procedure, angiographic equipment can deliver more radiation to the skin than most radiation therapy units deliver in a single treatment session. This development carries the risk of radiation-induced tissue reactions and stochastic effects for both patients and staff. Therefore, radiation safety in IR is crucial for patient care, occupational safety and quality assurance [81].

Occupational radiation exposure results predominantly from scattered radiation originating from the patient towards the medical staff. Scatter radiation levels in the proximity of the patient can be relatively high, even under routine working conditions. If protection tools and operational measures are not sufficiently utilised, radiation-induced lesions of the eye may occur after several years of work [82, 83].

Levels of procedural radiation exposure are affected by multiple factors. Some of them are beyond operator control, for example, the complexity of the performed procedure or the body mass index (BMI) of the patient. However, others can be partially controlled, such as the position of the medical staff relative to the patient, the X-ray equipment and acquisition technique, and the radiation protection tools used [82].

Occupational radiation protection requires the appropriate education and training of the interventional radiologist in the procedural technique, knowledge of radiation protection and the use and availability of protective tools and equipment. All actions to reduce patient dose will not only affect the patient dose, but also reduce the radiation dose for the staff. Not attempting to decrease patient dose is, therefore, equivalent to neglecting the radiation protection for the IR team [83].

One of the most effective ways to lower radiation dose to the patient and the staff during image-guided procedures is by decreasing exposure time. This is true for CT as well as fluoroscopy-guided procedures. Nonetheless, careful planning of the procedure, optimisation of imaging protocols, optimisation of imaging parameters, and training of staff are also essential measures for the avoidance of an excessive dose to patients [84].

Relevant steps to minimise patient and occupational radiation [81]:

- adapt tube settings (tube current, focal spot, filtration, exposure time and tube voltage) to patient size
- use frame rates as low as reasonable during fluoroscopy
- use collimation, preferably virtual (off fluoroscopy)
- keep imaging detectors as close to the patient as possible and maximise the distance between the patient and X-ray tube
- use geometric and electronic magnification only if necessary
- use lateral and oblique views only if necessary
- shield staff from any unnecessary radiation exposure using a variety of lead-equivalent shields, sheets, aprons, thyroid and sternum protectors and lead glasses
- know the equipment
- use road-map imaging or stored fluoroscopy loops
- use last image hold instead of a single shot
- avoid unnecessary cone-beam CT, long fluoroscopy and multiple runs
- minimise the use of digital subtraction angiographic (DSA) acquisitions as much as possible, use frame rates as low as reasonable
- keep a record of the patient dose (kerma area product (KAP) and cumulative air kerma (CAK) and skin dose)
- only operate the fluoroscope when necessary
- use optimised and adapted examination programmes

Routine evaluation of DICOM dose reports and real-time dosimetry are extremely helpful to optimise radiation protection of patients and staff during interventional procedures. The dose levels should be compared with the national diagnostic reference levels regularly. The interventional radiologist performing potentially high-dose procedures should inform patients about the risk of skin injuries. When obtaining informed consent (see “Medico-Legal Aspects of IR” section) an explanation of the probability, characteristics and risks of deterministic injury should be included in the consent discussion prior to the procedure [84].

While there are legal minimum standards for documenting radiation exposure, additional documentation of the estimated radiation dose received by patients is recommended. Corresponding information should be recorded in the patient’s medical record following the procedure. If the CAK at the reference point exceeds 3 Gy, provisions should be made for follow-up of those areas for the determination of radiation effects. In such circumstances there should be documentation in the medical record that the patient was advised of the potential for radiation injury to the skin and was given instructions for proper follow-up. The SIR–CIRSE Guidelines for patient radiation dose management recommend that follow-up should be performed if the CAK at the reference point exceeds 5 Gy. Independent from these recommendations, national legislation that may deviate from the CIRSE-SIR statements has to be followed [85].

During a complex interventional procedure, angiographic equipment can deliver more radiation to the skin than most radiation therapy units deliver in a single treatment session. Thus, monitoring and minimising radiation exposure to the patient and the staff is crucial. The interventional radiologist performing potentially high-dose procedures should inform patients about the risk of skin injuries. Meticulous documentation of radiation exposure from IR procedures is essential.

### Medico-Legal Aspects of IR

Errors in medicine are a not-so-uncommon news headline. Many of these headlines focus on malpractice. In daily routine, medical errors without negligence are even more common, often resulting in a lawsuit. Most commonly, the inability to present written informed consent remains as the only verifiably offence. This is unfortunate, as with patient-centred medicine, the patient has a legal and a moral right to actively participate in their own health care, and shared decision making is an essential component of patient-centred medicine. The goal of patient-centred consent is to help a patient make a thoughtful health care decision.

#### Informed Consent

Informed consent is not the simple act of having a patient sign a formal document; informed consent is the process in which a health care provider educates a patient about the risks, benefits, probability of success and alternatives to the proposed procedure. The patient must be competent to make a voluntary decision about whether or not to undergo a procedure. Informed consent is both an ethical and legal obligation of medical practitioners [86].

The information provided during the consent discussion should include the following: diagnosis and prognosis, purpose and nature of the proposed treatment, risks and potential complications, expected benefits or effects of the proposed treatment, the risks of not accepting the proposed treatment, any reasonable alternatives to the proposed treatment and their risks, complications and expected benefits or effects [87]. The fundamental principle of consent states that patients must be given sufficient information in a way that they can understand to enable them to exercise their right to make informed decisions about their care.

The patient’s consent should be obtained by the interventional radiologist performing the procedure. It may be delegated to a member of the IR team, given that they are competent in the particular treatment and alternative treatments [88]. The consent discussion has to happen outside the immediate environment of the procedural room, preferably, on a hospital ward or in an outpatient facility. Patients must be given sufficient time to read the consent form thoroughly before signing it. To ensure that consent is freely given, patients should be given sufficient time to consider their options and to discuss the procedure with family before deciding whether or not to proceed with a proposed treatment. The time allowed for consideration depends on the severity of the underlying disease, the complexity of a procedure and the associated risks. The physician should remind the patient that it is ultimately his or her decision whether or not to proceed with a proposed plan and that the procedure will not occur without the patient’s authorisation. If a patient is competent to give valid consent, they is also competent to refuse to give consent. A patient can withdraw consent at any time, even after signing a consent form [89].

There are some scenarios in which consent by the patient is not possible. In these situations, the physicians have to act in a manner anticipated as the best for the patient. These situations include [86]:

- Life-threatening emergencies:
  When life-saving treatment is required and the patient is unconscious, consent of the patient for these measures can be assumed. Nevertheless, the reasons for the treatment and its results should be clearly explained to the patient after the treatment is completed. The need for immediate intervention must be noted in the patient’s medical record, including situation details, the reason for immediate intervention and the reason for not obtaining consent [5].
- Incapacitated patients (e.g. due to severe mental health conditions):
  Each individual patient should be assessed in order to decide if they are capable of giving valid consent. In cases in which the patient is considered incapable of giving valid consent or unable to understand or retain information due to memory conditions such as dementia, the patient’s best interests should be discussed among the relevant clinical teams. The treatment should be discussed as much as possible with the patient and their immediate family. In the case of chronic incapability, in most countries, a legal guardian is needed to consent to an invasive procedure.
- Children:
  Minors may have appropriate decision-making capacity, but they do not have the legal empowerment to give informed consent. Therefore, parents or legal guardians are needed to give informed permission for diagnosis and treatment, preferably with the assent of the child whenever possible.

#### Malpractice and Medical Errors

All interventions involve risks, and IR physicians are exposed to a high risk of medico-legal litigations due to several factors: first, the surgical and procedure nature of IR and the problems intrinsic to the techniques used; second, the sporadic complications that can occur and, finally, the need to operate on severely ill patients with compromised clinical status. In addition, there are strict regulations regarding the use of radiation in patients (see “Radiation Protection” section).

The Institute of Medicine (IOM) differentiates between adverse events, complications, and medical errors [90, 91]:

- An adverse event is an injury caused by the treatment process rather than the patient’s underlying disease process.
- A complication is an unfavourable consequence of the patient’s disease process, an accident, or an adverse reaction that aggravates the original disease
- A medical error is a deviation from the expected norm, regardless of whether it results in any harm.

People usually file a lawsuit because of an unexpected bad outcome, but not every negative outcome rises to the level of medical malpractice, and most patients are not in a position to determine whether or not true malpractice has occurred. Medical malpractice is any act or omission by a physician during the treatment of a patient that deviates from accepted norms of practice in the medical community and causes an injury to the patient. Medical malpractice’s liability is normally based on the laws of negligence. To show that medical negligence occurred, the aggrieved patient must show that [11, 92]:

- a duty of professional care existed
- such duty was breached when the physician deviated from the standard of care (negligence)
- injury resulted from such breach
- such injury is measurable in damages that the court can use to calculate the redress owed to the plaintiff.

These legal elements of a medical malpractice case must be proven by the patient suing the doctor to the applicable standard of proof required by law.

Errors in medicine happen commonly, even when the physician performing the procedure is experienced and knowledgeable. Fortunately, most do not result in patient harm, and only a few errors constitute medical malpractice. The Institute of Medicine reported that 90% of medical errors result from systemic problems rather than individual factors [35]. A critical analysis of the different types of errors may help a radiologist undertake corrective measures and standardised interventional procedures with protocols to avoid future medico-legal issues. A quality management programme is an effective way to minimise the number of complications and medical errors (see “Quality Management in IR” section). Proper communication with the patient and other professionals (see “Communication” section) as well as an adequate informed consent process as integral parts of IR practice also help to minimise negative consequences and medico-legal issues from interventional procedures.

Informed consent is more than signing a consent form; it is the process of involving the patient in the decision-making process. It is mandatory not only for legal reasons—it also improves the quality of practice and helps to minimise medico-legal consequences for interventional radiologists.

Medico-legal, adverse event, complication and medical errors need to be differentiated. With appropriate patient selection, meticulous pre-procedural evaluation, obtaining the patient’s informed consent, and post-procedural follow-up, errors in IR can be kept low and malpractice can be avoided.

## Infrastructure for IR Clinics

### Infrastructure

Many interventional radiologists are intimidated by the notion of setting up an IR clinic, but it is not as difficult as it may seem [34, 93]. With the advent of electronic medical records, more and more business can be carried out online rather than by the use of traditional paper charts, tremendously reducing the amount of administrative infrastructure needed. If the interventional radiologist wants a dedicated IR clinic with dedicated nursing staff and to be treated in precisely the same way as other clinicians, that is an excellent model. Technically, it is much easier to start with a smaller model and evolve from there.

As reimbursement and cost structures vary greatly among the different European health care systems, the following case study for an IR clinic cannot be universal—however certain basic principles and challenges remain true across most environments:

- Office Space
  The need for actual physical space is limited, and the space can be almost anywhere. It does not need to be in the traditional outpatient clinic setting; in fact, it is often easier to have space somewhere in the radiology department. Subleasing space in a running private practice is another affordable and easy solution, particularly at the beginning. This also reduces the financial risk in comparison with renting an entire space individually.
- Staff
  Excellent staff is key to success. A receptionist answering the telephone, doing the scheduling, etc., is essential. Furthermore, a nurse or equivalent is mandatory to help with patient care.
- Time
  Depending on patient load, an interventional radiologist should allocate one to two half-days for consultation. New patients need around 30–45 minutes, while patients in for follow-up need around 20-30 minutes. Patients should never be scheduled parallel to interventions.
- Equipment
  A fully equipped office is needed (desk with telephone, three chairs, etc.), along with dedicated IT (at least a computer with working PACS and two monitors to be able to discuss images with patients), A Doppler machine, ideally an ultrasound machine, and typical standard medical equipment (examination couch, monitor for vital signs, etc.). Costs can be cut considerably when there is access to the imaging infrastructure of diagnostic colleagues.

Since the costs can vary considerably depending upon the country and the exact type of organisational model, it is impossible to provide examples of amounts of money for each situation. However, in all cases, a tailored business plan is mandatory before beginning. The following case study describes a functioning and affordable set-up for an IR clinic.

For the beginning (or even later on) it may be prudent to carry out a relevant part of IR clinics in or around the ultrasound department. The practical method for this is that the patient arrives, they check in at ultrasound, and then the IR receives a message to attend to the patient in the ultrasound suite, where there is a small waiting room with an office attached to it. In that office, there is a sphygmomanometer, a simple hand-held ankle brachial index (ABI) machine. Furthermore, the IR has access to a female healthcare assistant, if needed as a chaperone. This set-up suffices for taking a focussed history and performing a physical examination, thereby identifying the problem at hand.

This system can be further streamlined by the pre-selection of the appropriate patients. For instance, if the patient has peripheral arterial disease, the IR in this set-up will perform ABI measurements in the clinic themselves, and before the patient arrives there can be a CT or MR angiogram, the results of which will be available for the IR. With this small setup, ultrasound can be directly performed to assess the groins/brachial/radial arteries for potential access, and then IR and patient can have a very detailed discussion of what potential treatment options are available for that particular problem.

In a different patient, e.g. with a lung mass, there is no need for the ultrasound component of the examination. Instead, the patient requires a CT-guided biopsy. History and physical are by necessity brief, however, in these particular patients one will often obtain respiratory function tests; these could also be obtained in advance. The clinic can be used to come to the appropriate decision together with the patient.

Once the patient has been seen by the interventional radiologist, the IR dictates a letter, for example, by use of an online app on his phone. The letter should detail the history, physical examination, results at hand, etc. After printing, it then is sent to the referring clinician and/or GP, and in many cases, it should be copied to the patient as well.

A lot of tests and investigations can be controlled or directed by the interventional radiologist, but there are certainly patients for which access to other services is essential, for instance, if a pre-interventional anaesthetic assessment is needed for complex revascularisation or a TIPS procedure. An anaesthetic consultation should be used by interventional radiologists in the same way that any other clinical specialty would, and that requires the patient to attend the pre-operative anaesthetic clinic. Likewise, if the patient has poorly controlled diabetes and peripheral arterial disease, the IR should obtain an endocrinology consultation, and this may be scheduled in relation to or separate from any proposed procedure.

In essence, an IR clinic can be designed in an affordable way and many of the issues can easily be overcome. Lack of a dedicated space in which to house an “Interventional Radiology Clinic” should not be a limiting factor for any interventional radiologist.

### Organisation of an Inpatient Clinic

Inpatients being cared for by IR services will be either a) patients formally admitted under IR or b) consults from other services. If not already established, IRs should strive to attain admitting rights, so that patients may be accepted via direct referral from family practitioners and consultants from other hospitals. Ideally, all IR patients would be admitted to a specific IR ward [94], as there is a benefit from “concentrating” patients with similar care needs in the same ward. In the absence of a dedicated IR ward, ideally, a set number of beds should be “protected” for elective IR admissions. Time and energy expended in negotiating these rights with hospital management is a necessary and invaluable investment. In hospital systems where the “money follows the patient”, the hospital will see the benefit of offering IRs the ability to receive and admit their own patients. Simonetti et al. documented the financial benefits to the institution derived, in part, from the shorter admission pathway and length of stay [94].

Proportionally, most inpatients under IR care are still likely to be under the care of another service. When referred to such a patient, it is important to approach the consult as a surgeon would, and to and review the patient clinically, even when the indication appears straightforward. Use the opportunity to establish rapport, answer questions and allay fears, including talking to the patient’s relatives. This consult should be formally documented in the patient’s medical chart, outlining one’s findings and recommendations. Over time, this pattern of practice helps to eliminate the reputation as a technician and establishes a longitudinal model of care, especially if patients are followed up in an IR outpatient clinic after discharge.

There should be a clear mechanism for triggering a consult—ideally documented in an SOP. This SOP should include rules regarding urgency, e.g. for critical or urgent consults such as bleeding and sepsis. Voice or face-to-face requests should be required in these cases, whereas less urgent consults may be placed via the hospital’s established ordering system. There should be an administrative lead monitoring these requests with nursing or junior IR triage. These consults can be managed innovatively within the technological constraints of an institution; for example, in addition to the typical consult request forms, a dedicated mobile phone or pager may be used, or, where permitted by institutional policy, apps developed specifically for communication between medical teams. In general, hospital standards should be followed.

Where possible, there should be a rota within the IR team to ensure a responsive consult and ward service. Ideally, one individual (IR trainee or fellow) would be “on-call” during office hours for ward-based consults, who can liaise with the duty IR(s) if the patient’s condition is urgent or critical. A consultant IR should attend less urgent consults with a member of the team to function as a scribe. They should review the patient with a member of the nursing staff, read the patient’s notes, see and examine the patient, review investigations previously performed, and order more if necessary. As typically occurs with other services, the organisation of these additional investigations can be appropriately delegated to the referring team. The earlier in the day the consults take place, the better so that other investigations can be performed and the required procedure performed on the same day. The “consult round” may follow the day 1 post-op round.

Time for consults needs to be factored into IRs’ schedules. This activity may have a negative impact on the total procedure numbers, but some of this will be regained by avoiding lost time, as can happen when a patient is brought to the IR department for a procedure that is deemed inappropriate by the IR when they see and review the patient. Given the significant time commitment, these clinical duties should clearly form part of the IR’s official job description (Table 5) [46].

**Table 5:** Reasonable time allotment for clinical duties in IR

Number of procedures performed in department per year	Number of hours of clinical care generated per day
Less than 1000 procedures per year	1–2 h per day
1–3000 procedures	2–4 h/day
More than 3000 procedures	4–6 h/day

There should be a clear pathway and routine for the admission of inpatients. When staffing levels permit, a junior IR or fellow should perform the initial admission followed by a subsequent review by the senior IR. As for consults, wards should know who to call so that the patient may be attended to quickly. Where junior medical staff are not available, clinical assistants or specialist nurse practitioners can be invaluable. Pathways dependant on the level of care required should be clearly defined and protocols should be kept on the ward for ease of reference by the nursing staff.

Regardless of patient type, behaviour on the wards should be aimed at encouraging cooperation with nursing and supportive staff to achieve the best outcomes for patients. Professionalism is a given; however, especially at the outset, a friendly and approachable manner will help to establish good relationships and to ensure that staff feel free to share concerns about a patient’s condition [24]. Repeated exposure to IR patients will ensure that the nursing staff becomes familiar with the typical pre- and aftercare requirements. This familiarity should be supplemented with an element of education on each ward visit.

Post-procedure ward rounds are especially important to ensure the anticipated course, and that complications are detected promptly. Discharged patients should receive all necessary advice (including care of drains, etc., and who to call in the event of problems), prescriptions, absence from work certificates and so forth. If community pharmacies are likely to be closed, ensure the patient receives their first night’s dose of medications prior to leaving the hospital. If not being discharged the evening of the procedure, the discharge plan should be formulated and shared with the ward staff. The majority of IR patients overnighting in the hospital will be discharged on the first post-procedural day. Following discharge, a formal letter summarising the patient’s admission, procedures and inpatient course should be dictated. A copy should be placed in the formal medical record and copies sent to the referring physician and the family practitioner.

Time and energy expended in negotiating admission rights with hospital management is a necessary and invaluable investment. There should be a clear mechanism for triggering and attending to consults to ensure a timely service. Good relationships with ward staff are key to ensure the best standards of care and outcomes for IR patients. Clear instructions should be provided to the patient and their family practitioner at discharge.

### Organisation of an Outpatient Clinic

The physical space needed for an outpatient clinic is limited; however, some minimum standards need to be met. Like in any other specialty, an IR patient expects to be seen by appointment in a clearly defined office, ideally including a receptionist and waiting room, rather than somewhere in between IR procedures in a little back room without equipment or staff [6, 95]. Depending on the local situation, the IR outpatient care unit can be set up as an independent outpatient clinic or integrated into an existing structure, such as the vascular or oncology centre. In terms of increasing recognition of IR as a specialty, having a dedicated clinic with the interventional radiologist’s name on it is useful in so far as people see Dr. X, Consultation Endocrinologist and Dr. Y. Consultant Interventional Radiologist, etc. Probably the biggest aspect in terms of administration of an outpatient clinic is obtaining the time in one’s job specification for this.

The value of having an outpatient clinic in IR is often understated or deprecated. Many hospital administrations, and sometimes even diagnostic colleagues, would rather have the interventional radiologist reading plain films, covering CT, etc. [62]. This aspect of time needs to be built into any job plan, as it is an essential component for a successful IR team. Assuming the time is available, there should be almost daily access to an IR clinic in a busy IR practice. Depending on the size of the group, this set-up might be even bigger and more formalised. The same facilities need to be available for interventional radiologists as for any other type of clinician; this includes access to space, charts, health care assistants, chaperones, phones, computers, etc. It very much depends on the practice model that each individual interventional radiologist wishes to pursue, but most often, these issues can be overcome and many of the issues dealt with much more simply and efficiently by a sole practitioner or an appropriately trained secretary (see “Infrastructure” section).

The billing for IR outpatient services will depend on whether the interventional radiologist works in the private or public sector. In the private sector, the initial consultation fee is almost always payable by the patient on the day, and depending on the patient’s individual level of health insurance coverage, part of this consultation fee may be redeemable upon submission of receipts. Also applicable in the private sector is providing patients with the appropriate code/s for their potential procedure. It is of the utmost importance that the patient checks with their insurers prior to any planned admission so that they are aware of their coverage and of any shortfalls or excesses that may be payable. Once the patient is fully informed and agrees to a procedure, a claim form will be submitted to the hospital, which in turn will be submitted to the insurer for payment. Most of the administrative activities can be performed by an experienced secretary who knows the ins and outs of coding and billing. This is well recognised in the USA, where the SIR runs coding and billing seminars on a regular basis [46]. In Europe, this is much more difficult due to the variety of healthcare systems and models. However, the basic principles apply universally.

The most important factor for a successful IR outpatient clinic is to obtain the time for this activity. Most other issues can be overcome by lateral thinking. To become economically successful, a team member knowing the ins and outs of coding and billing is indispensable.

### Staffing and Organisation

The number of interventional radiologists within a practice is primarily determined by the number and diversity of the procedures performed, and by how clinical and outpatient care is organised. The nursing staff’s composition and size are also defined by the number of tasks for which they are responsible, with substantial (inter)national differences throughout Europe[96]. In some countries, the nursing staff cares for the patients in the pre-procedure and recovery rooms, through procedure scheduling, technical support, patient monitoring as well as sedation during the procedures, whereas in other countries, any of these tasks are outsourced to other departments. However, patient and personnel safety should always be the primary consideration in determining the size and composition of the IR team. It is only under that condition that a consistent and appropriate level of care is achieved in the IR suite [97].

#### IR Team

The provision of an IR service requires teamwork within radiology and other specialties. Staffing levels will reflect the size of the practice and (outpatient) responsibilities and be tailored for individual departments [98]. The IR team usually comprises radiologists, IR nurses, IR radiographers, clinical assistants, and managerial support. In the context of the ever-growing demand for minimally invasive, image-guided interventions, all team members must be committed to an efficient and effective IR service. All team members must have specific IR knowledge and should be able to rely on each other at all times. Therefore, both IR physicians and staff should primarily be assigned to the IR suites and not entrusted with other (diagnostic) tasks within the department.

#### On-Call Duties

IR is a widely recognised and essential clinical service in the management of emergency patients. Moreover, patients undergoing procedures during off-hours are often more critically ill than patients undergoing elective procedures during the daytime. It is, therefore, imperative that hospitals provide a 24/7 IR service with adequate physicians, staff and equipment. With a sufficient workforce, this service can be provided within an individual centre. If this is not the case, collaboration should be sought with surrounding institutes to set up a regional service with formal contractual agreements about mutual responsibilities and the practical setup. On-call duties may adversely affect the practical performance of IR physicians and staff. The increased workload can also lead to psychological complaints, ranging from stress symptoms to burnout and absenteeism. To provide a high-quality and sustainable on-call service, it is advisable to moderate the service frequency per person and to ensure adequate rest immediately after a shift.

Consistent, high-quality IR care can only be sustained with sufficient number of IR physicians and staff. On-call duties should be organised in a structured manner with a healthy balance between workload and rest.

### Communication

IR clinics represent the main instrument of communication between physicians and patients, as well as physician to physician. This is because IR practice is strongly linked to pre-procedural, intra-procedural, and post-procedural care. It has been reported that inadequate communication is among the most frequent causes of malpractice lawsuits in radiology [11]. Communication should entail speaking, listening and typing letters to both patients and referring physicians. In this setting, the most important thing is to understand what interventional diagnosis and treatment pathway is planned by the physician and what the patient expects from it.

#### Physician-to-Physician Communication

The interventional radiologist should set up a communication link with the referring clinician before meeting the patient. If there is a long-standing relationship between the two, it is still fundamental to speak about the specific case every time in order to avoid any misunderstandings or errors. Objects of communication should include the patient’s psychological profile, the current clinical issue and planned post-interventional management. The latter may take place at the referring clinician’s office, or the IR clinic, or both. It is always helpful to clarify these responsibilities beforehand.

Physician-to-physician communication can potentially also have a marketing role beyond the clinical one. Interventional radiologists may take advantage of making referring physicians aware of IR procedures, thus triggering an increasing number of referrals. For this reason, it is important to constantly give feedback to the referring physician regarding the outcome of any procedure and the level of satisfaction of the patient.

#### Physician-to-Patient Communication

Unfortunately, patients often do not know anything about IR procedures. A common misconception is to think of interventional radiologists as “key-hole surgeons”. It is important to speak to most patients in plain language, minimising the medical terms and using comparisons from the daily experience of the patients. This approach may change according to the level of background knowledge or culture of the patient [6]. If the conversation with the patient is with a partner or a relative present, it is advised to interact directly with the patient if they are capable of understanding. Otherwise, help from a relative is always welcome.

In general, most conversations follow an order; why, what and how the procedure is planned to be undertaken, and what are the benefits and the risks [34]. It is crucial to allow sufficient time for questions from the patient. When taking notes on the patient´s history, such as checking for allergies or drugs, the use of a checklist is advised to avoid oversights (see “Patient Evaluation and Preparation” section). The patient should not leave the interventional radiologist’s office without having understood the reason for and the type of planned treatment. Ideally, the patient should also receive formal contact information, such as a business card, in order to permit direct contact with the IR service in case of additional questions or logistic details. Independent from obtaining written informed consent, the initial physician to patient communication should take place well in advance of the planned procedure, allowing enough time to let the patient think about the benefits and implied risks. It must always be kept in mind that a full interview cannot be replaced with a quick conversation on the day of a procedure.

Appropriate communication with patients and physicians is the backbone of any IR clinic. Diligent communication of pre-procedural planning and follow-up to referring physicians is essential to maintain and increase patient referrals. Finally, patients should be addressed on a comprehensive level with a focus on the specific patient’s requirements.
